# Neddylation activated TRIM25 desensitizes triple-negative breast cancer to paclitaxel via TFEB-mediated autophagy

**DOI:** 10.1186/s13046-024-03085-w

**Published:** 2024-06-26

**Authors:** Bowen Zheng, Fengyuan Qian, Xuehui Wang, Yuying Wang, Baian Zhou, Lin Fang

**Affiliations:** Department of Breast and Thyroid Surgery, Shanghai Tenth People’s Hospital, School of Medicine, Tongji University, 301 Yanchang Middle Road, Shanghai, 200072 China

**Keywords:** Neddylation, TRIM25, TFEB, Autophagy, PTX resistance

## Abstract

**Background:**

Paclitaxel (PTX) treatment resistance is an important factor leading to poor prognosis in triple-negative breast cancer (TNBC), therefore there is an urgent need to identify new target for combination therapy. Neddylation is a post-translational process that introduces a ubiquitin-like protein called neural precursor cell expressed developmentally downregulated protein 8 (NEDD8). Previous studies have found that neddylation is activated in multiple tumors, but its relationship with PTX chemotherapy sensitivity has not been reported.

**Methods:**

Differences in UBC12 and NEDD8 expression levels between PTX-sensitive and PTX-insensitive TNBC tissues were validated using public databases and immunohistochemistry. The in vitro and in vivo functional experiments were used to observe the effect of neddylation inhibition combined with PTX therapy on tumor progression. Co-IP, western blot and PCR assays were used to investigate the molecular mechanisms. Molecular docking was used to simulate the protein binding of UBC12 and TRIM25. Molecular dynamics simulation was used to observe the changes in TRIM25 protein conformation.

**Results:**

We found that in TNBC that is insensitive to PTX, NEDD8 and NEDD8 conjugating enzyme UBC12 are highly expressed. Treatment with the NEDD8-activating enzyme (NAE) inhibitor mln4924 or knockdown of UBC12 significantly increased the sensitivity of the tumor to PTX, and this increase in sensitivity is related to UBC12-mediated autophagy activation. Mechanistically, UBC12 can transfer NEDD8 to E3 ubiquitin ligase tripartite motif containing 25 (TRIM25) at K117. Molecular dynamics simulations indicate that the neddylation modification of TRIM25 reduces steric hindrance in its RING domain, facilitating the binding of TRIM25 and ubiquitylated substrates. Subsequently, TRIM25 promotes the nuclear translocation of transcription factor EB (TFEB) and transcription of autophagy related genes by increasing K63-polyubiquitination of TFEB, thereby reducing tumor sensitivity to PTX.

**Conclusions:**

Neddylation is activated in PTX-insensitive TNBC. Specifically, autophagy gene transcriptional activation mediated by the UBC12/TRIM25/TFEB axis reduces TNBC sensitivity to PTX. Neddylation suppression combination with PTX treatment shows a synergistic anti-tumor effect.

**Supplementary Information:**

The online version contains supplementary material available at 10.1186/s13046-024-03085-w.

## Background

Triple-negative breast cancer refers to a type of breast cancer lacking the expression of estrogen receptor, progesterone receptor, and human epidermal growth factor receptor-2 [1]. TNBC represents the subtype with the poorest prognosis, posing a significant threat to women’s health [2]. At present, due to its unique molecular subtypes and lack of effective targets, chemotherapy, and surgery are the mainstays of treatment for TNBC. However, the development of resistance to chemotherapy and low rates of response (10–15%) frequently hinder the successfulness of the treatment [1]. Therefore, it is meaningful to seek new targets to sensitize chemotherapy.

Autophagy is a highly conserved eukaryotic biological process closely related to TNBC progression and chemotherapy resistance. Autophagosomes in tumor cells engulf damaged organelles and fuse with lysosomes to facilitate protein recycling, aiding in their survival under malignant conditions [3]. TNBC cells demonstrate higher levels of autophagy compared to other subtypes [4]. Previous research suggested that autophagy inhibition restored the sensitivity of TNBC cells to chemotherapy [5]. Therefore, autophagy is a potential target for the treatment of TNBC. TFEB, a transcriptional factor, plays a crucial role in regulating the autophagy-lysosomal system [6]. TFEB is associated with tumor progression and promotes various cancer phenotypes by regulating the autophagolysosomal system, even in an autophagy-independent manner [7]. In a state of abundant nutrition, TFEB undergoes high phosphorylation modification, and the phosphorylated amino acid sequence is recognized by 14-3-3 protein, leading to its cytoplasmic sequestration [8]. When external conditions change, such as cold, hunger, or lipopolysaccharide (LPS) treatment, TFEB rapidly dephosphorylates and translocates into the nucleus to respond to the stimuli [9]. However, there are still many unknowns regarding the regulatory role of other post-translational modification (PTM) on TFEB.

Neddylation is a PTM akin to ubiquitination that involves the transfer of the ubiquitin-like molecule NEDD8 to the target substrates. This process regulates the activity and function of the changed proteins. NEDD8 delivery follows the E1-E2-E3 rule [10]. The classical substrates for neddylation are cullin family proteins, with UBC12 and UBE2F as known NEDD8-delivering E2 enzymes [11]. Unlike UBE2F, UBC12 has been reported to mediate the neddylation of other family proteins such as p73 [12] and SHP2 [13]. Neddylation is activated and associated with malignant progression in a variety of tumors [14, 15], but its relationship with chemoresistance is unclear.

Here, we report that UBC12 induced the neddylation of E3 ubiquitin ligase TRIM25. Neddylation modification of TRIM25 reduces steric hindrance in its RING domain and increases TRIM25’s interaction with TFEB. Subsequently, TRIM25 promotes the nuclear translocation of TFEB and transcription of autophagy-related genes by increasing its K63-polyubiquitination, thereby reducing tumor sensitivity to PTX. Notably, treatment with the NEDD8-activating enzyme (NAE) inhibitor mln4924 or knockdown of UBC12 significantly increased the sensitivity of TNBC to PTX. Thus, we reveal that the NAE-UBC12-TRIM25-TFEB-autophagy axis is a novel mechanism regulating the transcription of autophagy-related genes in chemotherapy-insensitive TNBC and is critical for ameliorating PTX resistance. TRIM25 is a potential downstream target for treating chemotherapy-insensitive TNBC.

## Methods

### Tissue samples

Breast tumor tissues and the adjacent normal tissues from BC patients were collected by the Department of Breast and Thyroid Surgery at Shanghai Tenth People’s Hospital of Tongji University in Shanghai, China. For patients with primary breast cancer, after receiving 175mg/m^2^ PTX every two weeks for a total of 4 treatment cycles, or 80mg/m^2^ PTX treatment weekly for 12 consecutive weeks, if the tumor size does not significantly decrease, we classify them as PTX-resistant patients. The institutional ethics committees of Shanghai Tenth People’s Hospital approved the informed consent provided by each study participant. The procedures followed in this study were in compliance with the principles outlined in the Helsinki Declaration.

### Cell culture and transfection

HEK293T cell and the breast cancer cell lines MDA-MB-231, BT-549 were purchased from the Chinese Academy of Sciences (Shanghai, China). MDA-MB-231 and HEK293T cells were cultured in Dulbecco’s Modified Eagle’s Medium (DMEM) (from Gibco, USA) supplemented with 10% Fetal Bovine Serum (FBS) (from Gibco, USA), 100x penicillin and streptomycin mixture solution (from Enpromise, China). BT-549 cells were grown in the RPMI-1640 Medium (from Gibco, USA). The PTX-resistant MDA-MB-231 cell (MDA-MB-231-R) was gifted by Dr. Wei (from NIBS, Beijing, China), and was continuously cultured in DMEM complete medium containing 10nM PTX. All the aforementioned cell lines were cultured at 37 °C with a 5% CO_2_ atmosphere. Reagent Lipo8000 (from Beyotime, Shanghai, China) was used for transfecting different knockdown or overexpression plasmids into cells. First, mix the serum-free DMEM medium, Lipo8000, and plasmid according to the instructions of the reagent supplier. Then, add the mixture to the culture medium of cells with 80% confluence. After 8 h, replace it with fresh complete culture medium. All overexpression plasmids were obtained from MiaoLingBio (Wuhan, China), some of which were in stock. Knockdown plasmids (sh-NC, sh-TRIM25 and sh-UBC12) were kindly provided by Dr. You and Dr. Chen. The sh-TFEB plasmid was purchased from Generay Company (Shanghai, China).

### MTT, colony formation, wound healing, and transwell migration

Breast cancer cells were seeded in a 6-well plate and transfected with plasmids, mln4924, and PTX for 24 h of treatment. Then, cells were treated with trypsin, and the cell density was quantified to obtain the required number of cells for subsequent experiments. **MTT** 2000 cells were seeded into individual wells of 96-well plates. The cells were characterized following the guidelines provided by the manufacturer, using an MTT assay (from Sigma, California). The optical density at 490 nm was measured using a microplate reader after 24, 48, 72, and 96 h. **Colony formation** Six-well plates were used, and 1000 cells in total were transplanted into each well. We rinsed the colonies twice with cold PBS, stained the cells with 0.1% crystal violet, and preserved the cells in 75% ethanol to observe the colonies. The colonies were then counted and taken pictures. **Wound healing** Using a 200 µl pipette tip, a scratch was created in each well once the cells had reached approximately 90% confluency. The tip was maintained in alignment with the plate. Before being cultured in DMEM media without FBS, the monolayers were washed twice with 1x PBS to minimize the influence of cell proliferation on the results. A light microscope was employed to observe the identical area of the wound healing process. **Transwell migration** Cell migration within 24-well plates was assessed using transwell chambers (Corning, Inc., Lowell, MA, USA). 200 µl of serum-free media were utilized to transfer cells into the upper chamber, while a medium containing 10% FBS was introduced into the lower chamber. After 24 h, the cells in the upper chamber were removed with a cotton swab. Following this, the cells on the underside of the filter were stained with 0.1% crystal violet for 10 min and fixed with 70% ethanol for 30 min. Images were captured using a representative microscope.

### Animal experiments

The sh-UBC12, sh-NC, OE-TRIM25 WT, OE-TRIM25 K117R, and OE-vector stable MDA-MB-231 cell lines were acquired by screening with puromycin (from Beyotime, ST551). The plasmid we used to construct stable knockdown UBC12 cells was with pLKO.1 as the vector. 4-week-old female BALB/c nude mice were ordered from SLAC (Shanghai, China) and divided into several groups randomly (*n* = 4 each group). These cells were suspended in 150 µl of DMEM without fetal bovine serum and given to mice through subcutaneous injection. Each mouse was injected with 1.5 × 10^6^ cells. Three days later, tumor formation was observed and the mice were treated with PTX (10 mg/kg), which was pre-dissolved in PBS, via intraperitoneal injection once every 3 days, while the control group was injected with an equal volume of PBS. The mice were euthanized after 20 days, and the xenograft tumors were harvested.

### Cell drug intervention

Cells were exposed to 200ng/ml LPS (MCE, HY-D1056) for 4 h, and 20nM PTX (MCE, HY-B0015) for 24 h. For neddylation inhibition, cells were exposed to 100 nM MLN4924 (MCE, HY-70,062) for 24 h. To inhibit the fusion of autophagosomes and lysosomes, cells were treated with 25 μm Chloroquine (MCE, HY-17,589 A) for 24 h. To inhibit phosphorylation of mTOR, cells were treated with 10 μm MHY1485 (MCE, HY-B0795) for 4 h. To inhibit protein synthesis, cells were treated with 20ug/ml CHX (MCE, HY-12,320) for the appointed time.

### RT-PCR

TRIzol reagent (from Beyotime, Shanghai, China) was used to isolate total RNA from cells in accordance with the manufacturer’s instructions. Using a cDNA Synthesis Kit (from Abclonal, China), single stranded cDNA was created. The SYBR Green Master Mix (from Abclonal, Shanghai, China, RK21203) was used to perform qRT-PCR. The primers used are listed in Table S1.

### Protein separation

To extract the total proteins from the cells, phosphatase inhibitor (50X) and protease inhibitor (PMSF 100X) were added to the RIPA. The cells were allowed to lyse on ice following the addition of the mixture. After 30 min, the proteins were scraped off, and the supernatant was extracted using high-speed centrifugation. The supernatant and loading buffer combination were heated to 100 °C for ten minutes. Protein separation of cytoplasm and cytosol was performed following the instructions of the reagent vendor (Beyotime in Shanghai, China). Cells were lysed in a particular buffer, and the lysate was spun up for 5 min at 14,000 g/min. The supernatant constituted the cytoplasmic fraction, while the pellet (nuclear fraction) was lysed in RIPA buffer and sonicated. Soybean-sized tissue was cut with scissors, lysis buffer was added at 4 °C, and the tissue was completely homogenized to extract total proteins from patient-sourced samples. The subsequent steps followed the same protocol as for extracting proteins from cells. Complete the mitochondrial separation experiment according to the manufacturer’s instructions (Beyotime, C3601). After that, RIPA was used to extract the protein.

### Immunoprecipitation

The cells were lysed using Co-IP buffer including protease and phosphatase inhibitors. After 10-minute centrifugation at 12,000 g, the supernatants were collected and treated for an overnight period with protein A/G magnetic beads (from absin, abs995) and the antibodies at 4 °C. The next day, the beads were washed three times using the IP buffer. Elution was performed by boiling the immunoprecipitates in 1xSDS sample buffer.

### Western blot

SDS-PAGE was used to separate protein samples, which were then moved onto the nitrocellulose filter membrane using Rapid Transfer Buffer (from New Cell & Molecular Biotech, WB4600). After blocking with 5% BSA, the membrane was incubated overnight with primary antibody and then incubated with secondary antibody for 1 h at room temperature and washed the membrane with TBST for 10 min three times. Finally, the blots were exposed by chemiluminescence. The antibodies used are listed in Table S1.

### Silver staining and Mass spectrum

BT-549 cells were transfected with Flag-UBC12 or Flag-TFEB plasmids for 48 h and treated with MG132 (25 mM) for 3 h. Following, immunoprecipitation of the cell lysates was performed with antibodies against the Flag epitope tag. The Protein Silver Stain Kit (from Beyotime, P0017S), was utilized for silver staining, and the guidelines provided by the manufacturer were adhered to. Mass spectrum was used to identify UBC12 and TFEB interacting proteins separately.

### Immunofluorescence staining

BT-549 were cultured on glass coverslips in 24-well plates. Cells were fixed for 10 min, then permeabilized with 0.1% Triton X-100, blocked with 5% BSA for 30 min, then stained with particular antibodies. BT-549 cells transfected with the respective plasmids were stained with the appropriate anti-Flag and anti-Myc antibodies. Using secondary antibodies coupled with DyLight 488 (AS037, abclonal, China) and DyLight 594 (AS039, abclonal, China). Stain nuclei with DAPI. After taking pictures, the images were analyzed using ImageJ.

### Immunohistochemistry (IHC)

Patient tissue specimens and xenograft tumors were stained with anti-UBC12 (14520-1-AP, proteintech, China), anti-NEDD8 (16777-1-AP, proteintech, China), anti-KI67 (provided by Servicebio), and anti-LC3B (provided by Servicebio) after fixation, dehydration, embedding, and sectioning. After taking pictures, the images were analyzed using ImageJ. For each IHC slide, observe five fields (200X magnification) and calculate the percentage of positive cells. The IHC score is the product of the percentage of positive cells and the intensity of positive staining.

### Monodansyl cadaverine (MDC) staining

To assess autophagy, a fluorescent probe MDC was utilized as a specific indicator for acidic vesicular organelles such as autophagic vacuoles. In short, the transfected BT-549 cells cultured on coverslips were treated with MDC (from Beyotime, C3018S) for 40 min at 37 °C in the absence of light for staining. Following three washes with Assay Buffer, the cells were examined using a fluorescence microscope.

### JC-1 assays

Cells were seeded in 12-well plates and transfected with plasmids 24 h ahead of time. Followed by JC-1 experiments according to the reagent vendor’s instructions (Servicebio, China, G1515-100T) and photographed using an orthogonal fluorescence microscope.

### Molecular docking

To analyze the binding affinities and modes of interaction between UBC12 and TRIM25, AutoDockTools (https://autodocksuite.scripps.edu/adt/) was employed. The AlphaFold Protein Structure Database (https://alphafold.ebi.ac.uk/) was used to get the crystal structures of the ligand binding domain from human UBC12 (P61081) and TRIM25 (Q14258). To perform dock assays, all protein files were converted into the PDBQT format, removing all water molecules and adding polar hydrogen atoms. The grid box was positioned in the middle to permit free molecular mobility and include the domains of the protein.

### Molecular dynamics simulation

Homo sapiens TRIM25 and NEDD8 structures were obtained from AlphaFold Protein Structure Database. The structure of activated NEDD8 was obtained by first intercepting its tail sequence to expose the carboxyl group of glycine at site 76 [16]. Molecular dynamics simulations were conducted by GROMACS (https://www.gromacs.org/). Specifically, first, we used the Relax ScriptManager application to select the models of TRIM25 with the lowest energy scores for NEDD8 chemical conjugate docking. Lysine at site 117 of TRIM25 forms an isopeptide bond linkage with activated NEDD8-terminal glycine. However, due to the lack of fully validated force field data files to support, we only used NEDD8 as a ligand to observe the protein conformational changes after its spatial proximity to site 117 lysine of TRIM25. Then, each system was exposed to a 100ns MD simulation to assess the movements of the atoms and residues. Trajectory images were recorded every 1 ps. Last, using the UCSF Chimera57 software version, all of the molecular visuals were made.

### Statistical analysis

The data obtained from three separate experiments were depicted as the mean ± standard deviation. Statistical analysis was performed using GraphPad Prism 8 (GraphPad Software, San Diego, CA). One-way ANOVA was conducted, followed by Dunnett’s post hoc test, and two-way ANOVA was followed by Tukey’s post hoc test. The significance threshold for each analysis was set at *p* < 0.05.

## Results

### Inhibiting the neddylation signal increases TNBC sensitivity to PTX

**Fig. 1:**
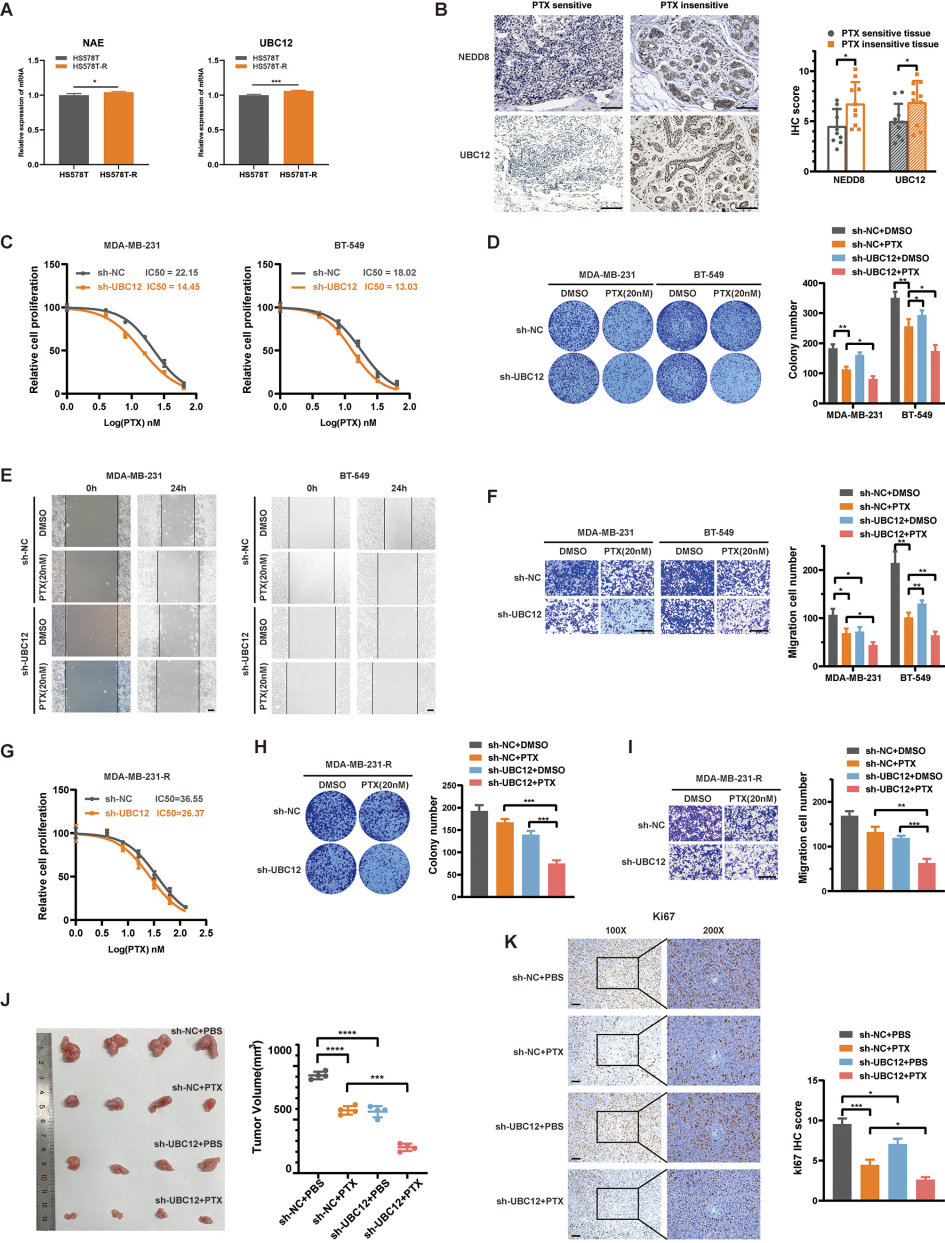
Inhibiting neddylation increases TNBC sensitivity to PTX. **A** NAE and UBC12 mRNA expression in normal and chemotherapy-resistant cells (HS578T-R). **B** IHC assays of PTX sensitive and insensitive TNBC tissues. Representative images were shown. Scale bars, 100 μm. **C** After being transfected for 24 h, TNBC cells were treated with a serial dose of PTX for 24 h and subjected to MTT assay. The IC50 values of PTX in each group were indicated. **D-F** Colony formation, wound healing and transwell migration assays were used to assess the proliferation and migration capacity of the cells. Scale bars, 200 μm. **G-I** MTT, colony formation and transwell migration assays were used to assess the proliferation and migration capacity of the MDA-MB-231-R cells. Scale bars, 200 μm. **J** MDA-MB-231 cells stably expressing sh-NC and sh-UBC12 were subcutaneously injected into the nude mice. These mice were treated with or without PTX. Tumor images and volumes were shown. **K** IHC assays of KI67 protein in xenograft tumors. Scale bars, 100 μm. Data are presented as mean ± SEM. *p < 0.05, **p < 0.01, ***p < 0.001, ****p < 0.0001

Neddylation has been reported to be activated in a variety of tumors [14, 17], and we noted higher expression of UBC12 (E2 for NEDD8 transfer) in breast cancer tissues compared to normal tissues in The Cancer Genome Atlas (TCGA) database (Fig. S1A). Moreover, the high expression of UBC12 is associated with poor prognosis in breast cancer (Fig. S1B). Consistently, high expression of UBC12 protein was observed in the paired breast cancer samples we collected (Fig. S1C). However, the relationship between neddylation and chemotherapy resistance remains unknown. In the GSE144113 dataset, we noted elevated expression of NAE and UBC12 in chemotherapy-resistant HS578T cells induced by repeated low-dose PTX stimulation (Fig. 1A). We collected ten tissue samples each from TNBC patients who were sensitive or insensitive to PTX treatment, and immunohistochemical (IHC) analysis indicated high expression of NEDD8 and UBC12 in tissues with poor PTX chemotherapy sensitivity (Fig. 1B). These observations indicated a correlation between neddylation activation and PTX insensitivity. By targeting the crucial E2 UBC12 in NEDD8 signaling, we observed increased PTX sensitivity upon UBC12 knockdown in vitro, manifesting as a significant decrease in IC50 values in MTT assays (Fig. 1C). Furthermore, co-treatment of UBC12 knockdown and PTX therapy was validated to synergistically inhibit TNBC cell proliferation and migration in colony formation, wound healing, and transwell migration assays (Fig. 1D-F). In addition, experiments were also conducted in MDA-MB-231 cells resistant to PTX (MDA-MB-231-R). Its PTX resistance was confirmed through MTT analysis (Fig. S1D). Consistently, knocking down UBC12 partially restored the sensitivity of resistant cells to PTX (Fig. 1G-I). In subcutaneous xenograft tumors of nude mice, we observed a significant reduction in tumor volume as well as a decrease in the percentage of proliferative antigen KI67 positive cell counts in the tumor tissue after combination therapy (Fig. 1J and K). In contrast, overexpression of UBC12 promotes cell proliferation and migration (Fig. S1E, F). We also utilized the NAE’s (NEDD8 activating enzyme) inhibitor mln4924 in combination with PTX treatment in cells [18], and similarly observed a good synergistic effect in suppressing the malignant phenotype of TNBC (Fig. S1G-J). Our results indicate that the neddylation is activated in PTX-insensitive TNBC, and sensitizing the cells to PTX by blocking neddylation signal is feasible.

### UBC12 promotes autophagy in TNBC

**Fig. 2:**
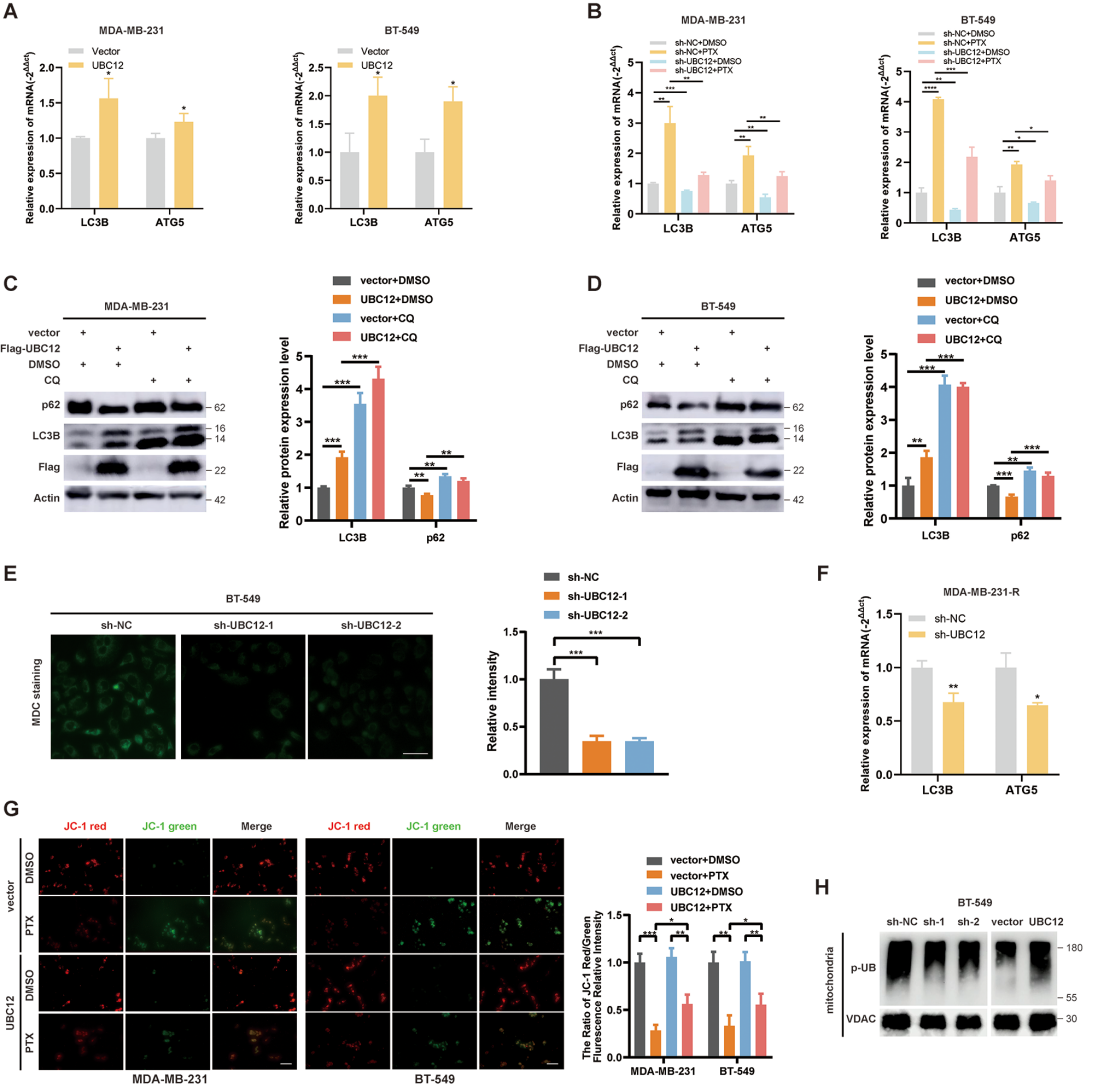
UBC12 promotes autophagy. **A** and **B** PCR assays of expression level of LC3B and ATG5 mRNAs. **C and D** Western blot analysis of the expression level of LC3B and p62 proteins when UBC12 was overexpressed, or autophagy inhibitor CQ was used. **E** MDC staining assays of acidic vesicular organelles formation when UBC12 was knocked down. Scale bars, 50 μm **F** PCR assays of expression level of LC3B and ATG5 mRNAs when UBC12 was knocked down in MDA-MB-231-R cell. **G** Mitochondrial membrane potential was detected by JC-1 assays when UBC12 was knocked down. Scale bars, 50 μm. **H** Western blot analysis of protein level of phosphorylated ubiquitin in mitochondrial fractions. Data are presented as mean ± SEM. *p < 0.05, **p < 0.01, ***p < 0.001, ****p < 0.0001

Next, we investigated how the neddylation signal affected the sensitivity of PTX. We noticed that PTX has been reported as an inducer of autophagy [19]. Autophagy is a conserved cellular process related to chemoresistance in breast cancer [19]. We conjectured whether neddylation was associated with autophagy activation. In the RNA-seq dataset GSE222110, we noted a downregulation of the autophagy-related gene MAP1LC3B2 transcription in HEK293T cells following siRNA targeting E2 treatment (Fig. S1K). Subsequently, we overexpressed UBC12 in TNBC cells and observed transcriptional activation of autophagy genes LC3B and ATG5 as expected (Fig. 2A). On the contrary, knockdown of UBC12 attenuated PTX-induced autophagy activation (Fig. 2B). From western blot assays, we found that UBC12 overexpression could upregulate the protein expression of LC3B but downregulate the protein expression of SQSTM1 (p62) (Fig. 2C and D). Additionally, chloroquine (CQ, inhibitors of autophagic vesicle and lysosomal fusion [20]) treatment could inhibit the autophagic flux mediated by UBC12 which was manifested by the accumulation of LC3B- II and p62. (Fig. 2C and D). After knockdown of UBC12 using shRNA, we performed Monodansyl cadaverine (MDC) staining and observed the suppression of acidic vesicular organelle formation, including autophagic vacuoles (Fig. 2E). In addition, inhibition of autophagy gene transcription was observed in PTX-resistant cell upon UBC12 silencing (Fig. 2F). We also examined the effect of UBC12 on cellular mitophagy under PTX stress. Overexpression of UBC12 partly alleviated the decrease in mitochondrial membrane potential caused by PTX treatment (Fig. 2G). Additionally, overexpression of UBC12 upregulated the level of phosphorylated ubiquitin(an indicator of mitophagy level [21]) in mitochondrial components, downregulation was observed when knocking down UBC12 (Fig. 2H), suggesting that UBC12 might promote mitophagy to clear damaged mitochondria. These results show that UBC12 promotes autophagy in TNBC cells and that its role is broad, involving both macroautophagy and mitophagy.

### The activation of autophagy by UBC12 depends on the nuclear translocation of TFEB

**Fig. 3:**
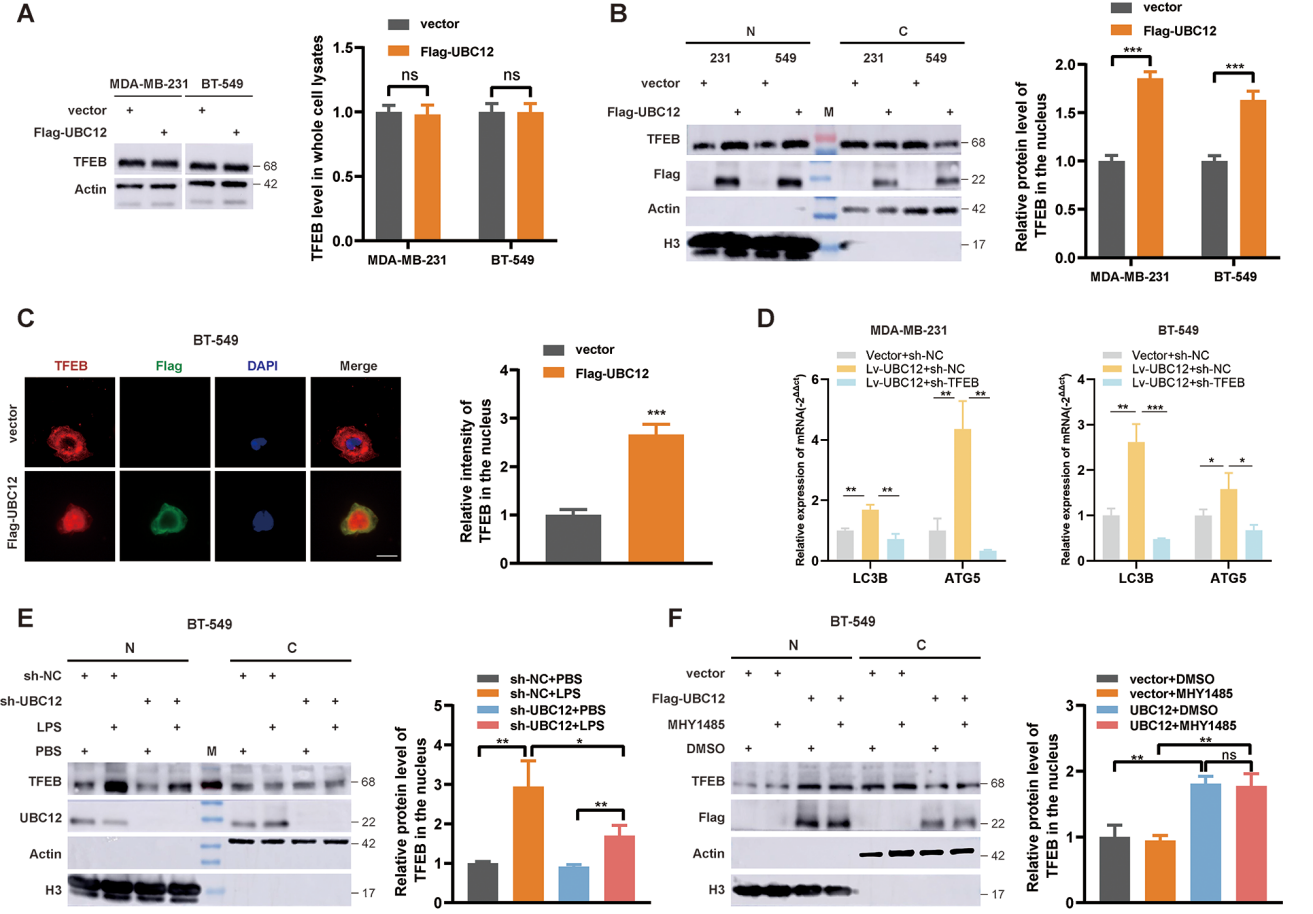
UBC12 induces TFEB nuclear translocation. **A** Western blot analysis of the expression level of TFEB proteins in whole cell lysate. The right-hand side showed the statistical graph after quantification, and the same below. **B** Western blot analysis of the expression level of TFEB proteins in nucleus (N) and cytoplasm (C). **C** IF analysis of the location of UBC12 and TFEB in BT-549 cells. Scale bars, 20 μm. **D** PCR assays of expression level of LC3B and ATG5 mRNAs. **E** Western blot analysis of the expression level of TFEB in nucleus and cytoplasm when UBC12 was knocked down, with or without LPS treatment. **F** Western blot analysis of the expression level of TFEB in nucleus and cytoplasm when UBC12 was overexpressed, with or without mTOR agonist MHY1485 treatment. Data are presented as mean ± SEM. *p < 0.05, **p < 0.01, ***p < 0.001, ****p < 0.0001

Given the extensive impact of UBC12 on autophagy we observed, we speculated whether UBC12 regulated critical transcription factors to promote comprehensive transcription of autophagic genes. The MiT-TFE family proteins (including TFEB, TFE3, TFEC, and MITF) play crucial roles in lysosomal biogenesis and autophagy regulation [8]. We isolated and detected proteins in the cytoplasm and nucleus after transfecting UBC12 plasmid, and found that UBC12 could promote the nuclear translocation of TFEB, but does not affect its total protein expression (Fig. 3A and B). Additionally, we did not observe a significant regulatory effect on the same family protein TFE3 (Fig. S1L). In addition, immunofluorescence (IF) analysis showed that UBC12 increased the abundance of TFEB in nuclear fractions of BT-549 cells (Fig. 3C). Furthermore, knockdown of TFEB inhibited UBC12 overexpression-mediated transcriptional upregulation of LC3B and ATG5 (Fig. 3D). Due to the few distribution of TFEB in the nucleus under non-stress conditions, we did not observe the inhibition of TFEB nuclear translocation with UBC12 knockdown in the control group, but knocking down UBC12 attenuates nuclear translocation of TFEB under LPS stimulation (Fig. 3E). These results indicate that UBC12-mediated autophagy gene transcription activation depends on TFEB nuclear translocation. Previous studies have shown that TFEB nucleocytoplasmic distribution is mainly regulated by its phosphorylation status. Under non-stress conditions, TFEB 211 site serine is highly phosphorylated by mTOR and recognized by 14-3-3 protein, leading to cytoplasmic sequestration [22]. We treated cells with MHY1485 (an agonist of mTOR phosphorylation [23]) and did not observe inhibition of UBC12-mediated TFEB nuclear translocation (Fig. 3F). TFEB 212 site cysteine affects the recognition of TFEB S211 phosphorylation by 14-3-3 protein [24], and we observed that the TFEB C212S mutation partially attenuated UBC12-mediated nuclear translocation of TFEB (Fig. S1M). These results suggest that UBC12-mediated TFEB nuclear translocation is not dependent on its phosphorylation status.

### UBC12-mediated TFEB nuclear translocation is dependent on TRIM25

**Fig. 4:**
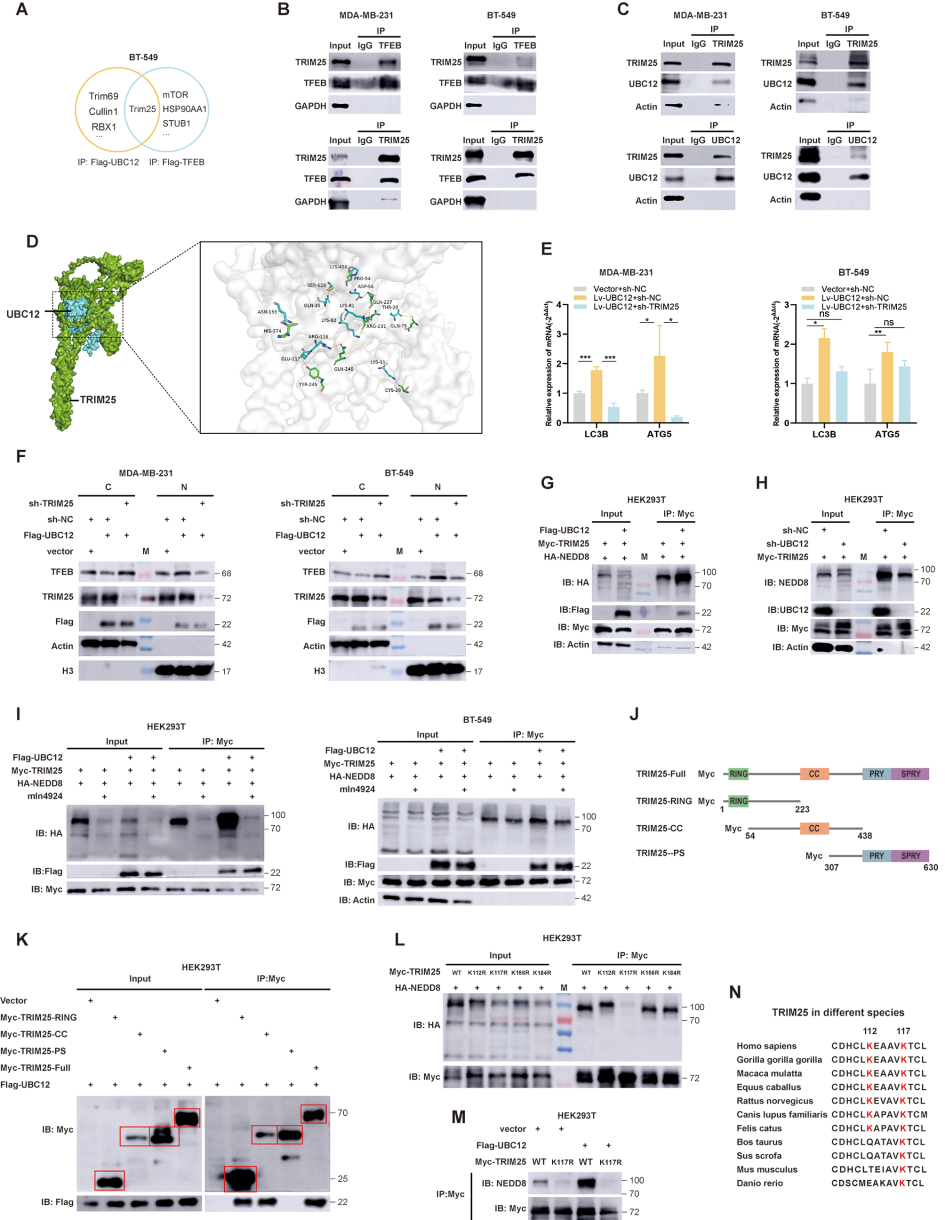
TRIM25 is neddylation at K117. **A** Venn diagram showing the Ub-E3 immunoprecipitated by Flag-UBC12 and Flag-TFEB as identified using MS. **B and C** CoIP and WB analysis of TRIM25, UBC12, and TFEB. **D** Molecular docking of UBC12 and TRIM25 using autodock software. **E** PCR assays of expression level of LC3B and ATG5 mRNAs after 24 h transfection of the plasmid. **F** Western blot analysis of the expression level of TFEB in nucleus and cytoplasm. **G and H** Western blot analysis of neddylation level of TRIM25 when UBC12 was overexpression or knocked down. **I** Western blot analysis of neddylation level of TRIM25 when UBC12 was overexpression, with or without mln4924 treatment. **J** Plasmid schematic. **K** Western blot analysis of UBC12 and segmented TRIM25. **L and M** Western blot analysis of neddylation level of TRIM25 when transfected with specific plasmids. **N** Conservation analysis of TRIM25 protein sequences in different species. Data are presented as mean ± SEM. *p < 0.05, **p < 0.01, ***p < 0.001, ****p < 0.0001

Given that UBC12 has been widely reported as the E2 for NEDD8 transfer [15, 17], we speculated whether UBC12 neddylate TFEB to regulate its nuclear translocation. However, we did not observe significant neddylation modification of TFEB in tumor cells (Fig. S2A), indicating that UBC12 does not directly regulate TFEB. Since the classic substrates for neddylation modification are the Cullin family proteins [25], which have not been reported to regulate TFEB nuclear entry, we speculated that the regulation of TFEB by UBC12 depends on an unknown E3 ubiquitin ligase. To identify the Ub-E3 that affects TFEB nuclear translocation, we immunoprecipitated UBC12 or TFEB from lysates of BT-549 cells overexpressing UBC12 or TFEB, respectively. Then, we take the samples to perform silver-stained and MS analysis (Fig. S2B). We found that in addition to the Ub-E3s that have been reported to bind to TFEB and UBC12 [15, 26, 27], TRIM25 was the only Ub-E3 detected in both samples (Fig. 4A). Furthermore, co-IP experiments also validated the endogenous interactions of TRIM25 with UBC12 and TFEB in breast cancer cells (Fig. 4B and C). The molecular docking suggested a good fit between TRIM25 and UBC12 in terms of conformation, with many covalent bonds or salt bridge interactions between the amino acids of the proteins (Fig. 4D), which further validate the physical binding of TRIM25 and UBC12. Additionally, knocking down TRIM25 abolishes the UBC12 overexpression mediated autophagic gene transcription activation and TFEB nuclear translocation (Fig. 4E and F), suggesting UBC12-mediated TFEB nuclear translocation is dependent on TRIM25. However, neither knockdown nor overexpression of UBC12 affected TRIM25 protein levels (Fig. S2C). Considering UBC12’s role in NEDD8 transfer, we speculated whether UBC12 mediates an increase in TRIM25 neddylation. We enriched exogenous TRIM25 with a Myc-tag antibody and subsequently detected exogenous NEDD8 with the HA antibody. In the IP fraction, we observed a clear band at a position (near 100kd) corresponding to a molecular weight greater than that of TRIM25 (near 70kd), suggesting the presence of neddylation modification in TRIM25. As anticipated, we observed an increase in the neddylation of TRIM25 in HEK293T cells upon overexpression of UBC12. Conversely, knocking down UBC12 resulted in a decreased neddylation of TRIM25 (Fig. 4G and H). Subsequently, we performed rescue experiments using mln4924, following treatment, a significant reduction in the neddylation of TRIM25 was observed, regardless of whether transfecting the empty vector or overexpressing UBC12 (Fig. 4I). According to reports, NEDD8 forms an isopeptide bond with the lysine residues of proteins through the C-terminal free carboxyl group, thereby conjugating to substrates [15]. To identify the exact site of TRIM25 neddylation, we constructed the plasmids with a Myc-tagged TRIM25 truncation sequence (Fig. 4J) based on previous studies [28]. The results of co-IP suggested that both the TRIM25-RING and TRIM25-CC truncations interact with UBC12 (Fig. 4K and Fig. S2D). Considering that protein neddylation is mostly found on adjacent lysine residues (for example, K358 and K364 in SHP2 [13], K370, K372, and k373 in p53 [29]), we ruled out the existence of distantly adjacent neddylation sites. Therefore, we narrowed down the range to 54–233 amino acids, which was consistent with the results of molecular docking (Fig. 4D). The in silico tool NeddyPreddy was used to predict neddylation sites (Fig. S2E) [30]. We mutated the top four lysine to arginine with higher scores in the sequence 54–233 and constructed new plasmids carrying Myc-tag (K112R, K117R, K156R, K184R). Subsequently, the mutant plasmids were transfected and the neddylation levels of TRIM25 were examined. We observed a significant decrease in the neddylation of TRIM25 only when the K117R mutation was present (Fig. 4L and Fig. S2F), and overexpression of UBC12 also failed to increase the neddylation level of the K117R mutated TRIM25 (Fig. 4M). For the adjacent K112, we speculated that its role in neddylation modification was relatively minor, possibly responsible for maintaining the NEDD8 conformation [16]. We also analysed the conservation of K112 and K117 in TRIM25 across species and found that K117 is highly conserved, while K112 is only conserved in higher animals (Fig. 4N). These results indicate that UBC12 increases the neddylation of TRIM25 at K117, and UBC12-mediated TFEB nuclear translocation depends on TRIM25.

### Neddylation is essential for TRIM25 regulation of autophagy

**Fig. 5:**
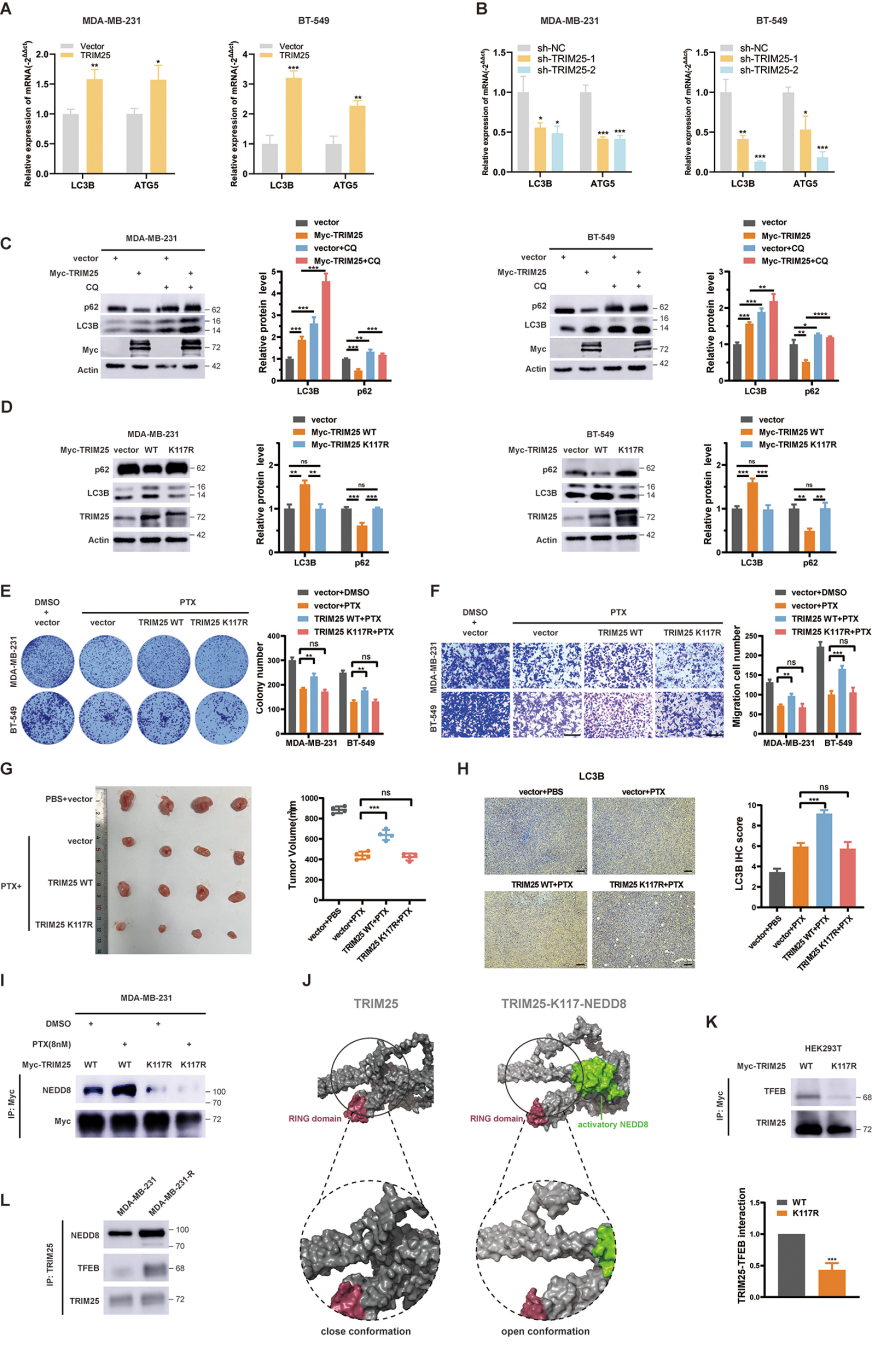
Neddylation is necessary for TRIM25 activation and autophagy regulation. **A** and **B** PCR assays of expression level of LC3B and ATG5 mRNAs when TRIM25 was overexpression or knocked down. **C** Western blot analysis of the expression level of LC3B and p62 proteins when TRIM25 was overexpressed, or autophagy inhibitor CQ was used. **D** Western blot analysis of LC3B and p62 when transfected with TRIM25 wild-type or mutant plasmids **E and F** Colony formation and transwell migration assays of two TNBC cells. Scale bars, 200 μm. **G** Tumor images and volumes were shown. **H** IHC assays of LC3B protein in xenograft tumors. Scale bars, 100 μm. **I** Western blot analysis of neddylation level of TRIM25 after stimulation with low concentrations of PTX. **J** Molecular dynamics simulations of TRIM25, with or without NEDD8 import. **K** Western blot analysis of interactive intensity between TRIM25 and TFEB. **L** Western blot analysis of TRIM25 neddylation level and TRIM25-TFEB binding. Data are presented as mean ± SEM. *p < 0.05, **p < 0.01, ***p < 0.001, ****p < 0.0001

Since fewer studies have suggested a direct relationship between TRIM25 and autophagy, we first analyzed their correlation in the TCGA database. We observed a positive correlation between the expression of TRIM25 and several autophagy related genes like ATG5, ATG7, and Atg12 (Fig. S3A). Furthermore, these genes are transcriptionally regulated by TFEB and are associated with poor prognosis in breast cancer [6] (Fig. S3B). From RT-PCR assays, we found overexpression of TRIM25 increased the transcription of ATG5 and LC3B, whereas knockdown of TRIM25 suppressed their transcription (Fig. 5A and B). Western blot results also indicated that TRIM25 overexpression activated autophagy flux (Fig. 5C). However, compared with the transfection of wild-type TRIM25 plasmid, no obvious autophagy flow activation was detected after transfection with the TRIM25 K117R mutant plasmid (Fig. 5D). In addition, an increase in autophagic vacuoles formation was not observed in MDC staining after overexpression of TRIM25 K117R (Fig. S3C). Colony formation, transwell migration assays, and xenograft tumor model demonstrated that overexpression of wild-type TRIM25 promoted PTX chemoresistance in TNBC both in vitro and in vivo, while overexpression of TRIM25 K117R had no effect (Fig. 5F-G). IHC analysis revealed an increase in the protein abundance of LC3B in xenograft tissues overexpressing wild-type TRIM25, but no significant changes were observed in tissues overexpressing TRIM25 K117R (Fig. 5H). These results indicate that TRIM25 reduces TNBC chemotherapy sensitivity by promoting autophagy, and neddylation is crucial for the activation of TRIM25. Interestingly, we also observed an increase in the neddylation of TRIM25 in wild-type MDA-MB-231 cells rather than TRIM25 K117R mutant cell after repeated low-dose PTX stimulation (Fig. 5I), which might represent another potential mechanism for PTX-induced autophagy.

### Neddylation activates TRIM25 via conformational change

Previous studies have found that neddylation affects protein function in various ways, such as inhibiting transcriptional activity [29], influencing phosphorylation activation [13, 31], and altering protein conformation to open the active site [32]. However, the specific impact of neddylation on TRIM25 function remains unclear. We carried out the following explorations. First, we detected that the K117R mutation had no significant impact on the subcellular distribution of TRIM25 (Fig. S3D). Then, we noticed two highly confident phosphorylation sites near K117, namely T91 and S100 (Fig. S3E). We enriched TRIM25 with Myc-tag antibody and detected phosphorylated serine and threonine levels, but no significant changes were observed (Fig. S3F). Last, we performed molecular dynamics simulations for TRIM25 and activated NEDD8 sequences. We observed no significant displacement in the RING structural domain, the primary active structure for TRIM25’s function as an E3 ubiquitin ligase [28], upon NEDD8 access K117 of TRIM25 (Fig. 5J). However, the protein arm located above the RING structural domain experienced a notable displacement, resembling the transition from a “clenched fist” to an “open hand” (Fig. 5J). This conformational change may reduce the steric hindrance of the RING domain, facilitating the entry of large substrates and subsequent ubiquitin transfer. Subsequent molecular biology experiments also confirmed that the K117R mutation weakens the binding of TRIM25 and its substrate TFEB (Fig. 5K). These data demonstrate that neddylation modification activates TRIM25 by altering its conformation. Interestingly, we observed a higher TRIM25 neddylation level and a stronger TRIM25-TFEB binding in PTX-resistant MDA-MB-231 cells (Fig. 5L).

### TRIM25 induces TFEB K63-linked polyubiquitination and nuclear translocation

**Fig. 6:**
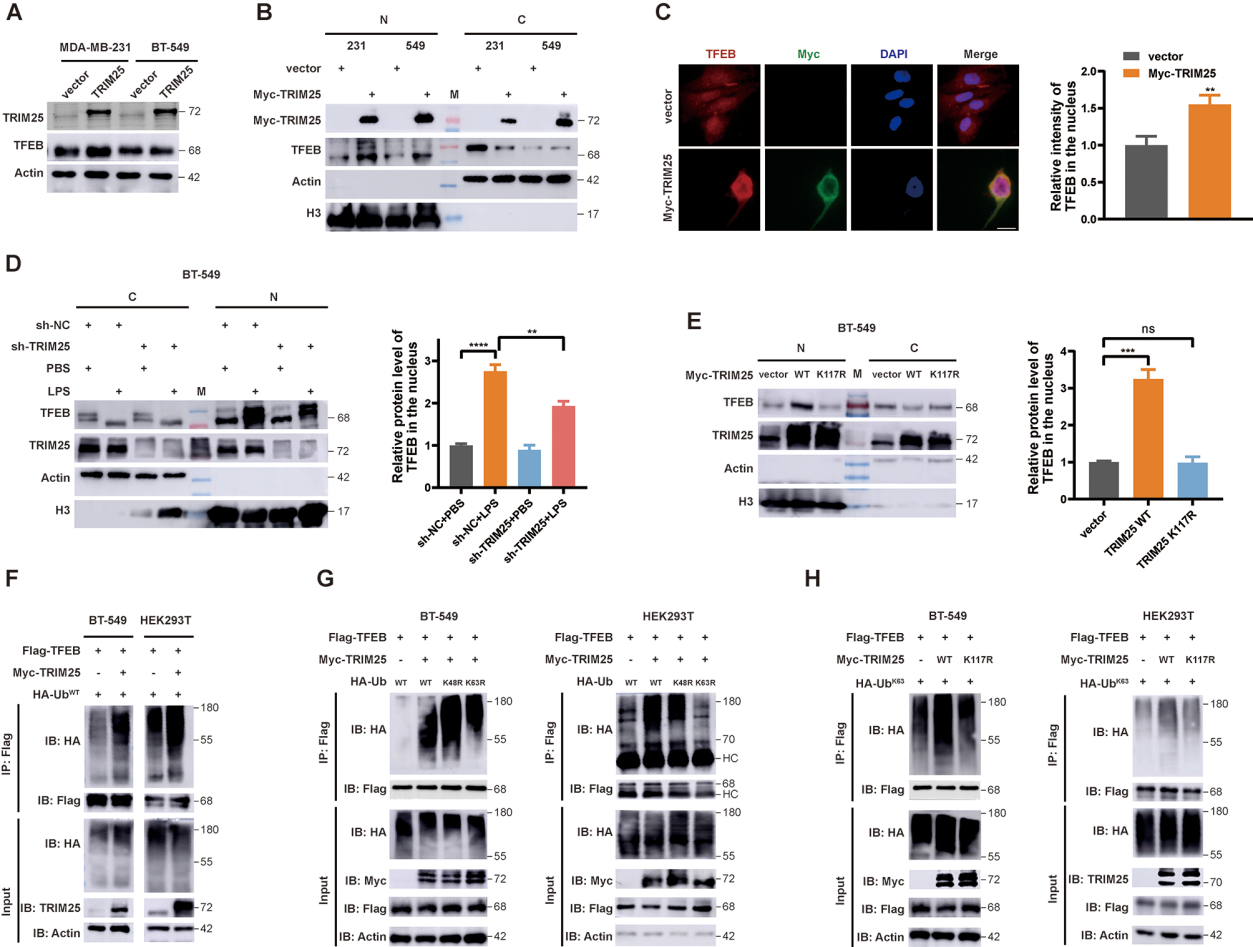
TRIM25 induces TFEB K63-linked polyubiquitination and nuclear translocation. **A** Western blot analysis of TFEB proteins in whole cell lysate. **B** Western blot analysis of TFEB proteins in nucleus and cytoplasm when TRIM25 was overexpression. **C** IF analysis of the location of TRIM25 and TFEB in BT-549 cells. Scale bars, 20 μm. **D** Western blot analysis of TFEB protein in nucleus and cytoplasm when TRIM25 was knocked down, with or without LPS treatment. **E** Western blot analysis of TFEB proteins in nucleus and cytoplasm when transfected with TRIM25 wild-type or K117R mutant plasmids. **F-H** Western blot analysis of ubiquitination level of TFEB when transfected with specific plasmids. Data are presented as mean ± SEM. *p < 0.05, **p < 0.01, ***p < 0.001, ****p < 0.0001

Although we validated the interaction between TRIM25 and TFEB, and the promotion of autophagy by TRIM25, the specific regulatory mechanism of TRIM25 on TFEB was still unclear. We found the overexpression of TRIM25 did not affect the total protein level of TFEB (Fig. 6A), but promoted the nuclear translocation of TFEB (Fig. 6B), which was consistent with the role of its upstream protein UBC12. IF assays confirmed that the overexpression of TRIM25 increased the abundance of TFEB in the nucleus as well (Fig. 6C). Under LPS stress, we found that TRIM25 knockdown reduced the abundance of the nuclear component TFEB (Fig. 6D). Moreover, transfecting TRIM25 K117R mutant plasmid did not induce TFEB nuclear translocation (Fig. 6E). We noted that E3s were reported to induce protein nuclear localization in ubiquitin-dependent manner [33], and this effect was commonly achieved through K63 polyubiquitin chains that did not affect protein stability [34]. We found that overexpression of TRIM25 increased the ubiquitination level of exogenous TFEB in cells (Fig. 6F). Polyubiquitination takes place at six distinct lysine residues within Ub molecule [35]. In search of specific types of TRIM25-mediated TFEB ubiquitination modifications, we employed two ubiquitin mutants where one lysine residue was substituted with arginine (K48R and K63R). We found that the ubiquitination of only the K63R mutant was hampered, providing evidence for TRIM25-mediated K63-polyubiquitination of TFEB (Fig. 6G). Furthermore, we observed overexpression of TRIM25 WT, rather than the neddylation-deficient TRIM25 K117R, leading to increased K63 ubiquitination of TFEB (Fig. 6H). Collectively, K63 ubiquitin chains on TFEB are enhanced in OE-TRIM25 WT cells, in parallel with its nuclear translocation. Moreover, the neddylation of TRIM25 is essential for TFEB ubiquitination modification.

TRIM25 was reported could reduce the stability of 14-3-3 [36], which is a key protein for recognizing phosphorylated TFEB [37, 38]. In BT-549 cells, we also observed that overexpression of TRIM25 WT, rather than K117R, reduced the protein levels of 14-3-3 (Fig. S3G, H). Overexpression of UBC12 shortened the half-life of 14-3-3 protein, but did not affect TFEB (Fig. S3I, J). This suggests that TRIM25-mediated 14-3-3 protein degradation may weaken the recognition and cytoplasmic sequestration of phosphorylated TFEB, providing another potential mechanism for inducing TFEB nuclear transport. Of course, this molecular mechanism requires more evidence to support.

## Discussion

**Fig. 7:**
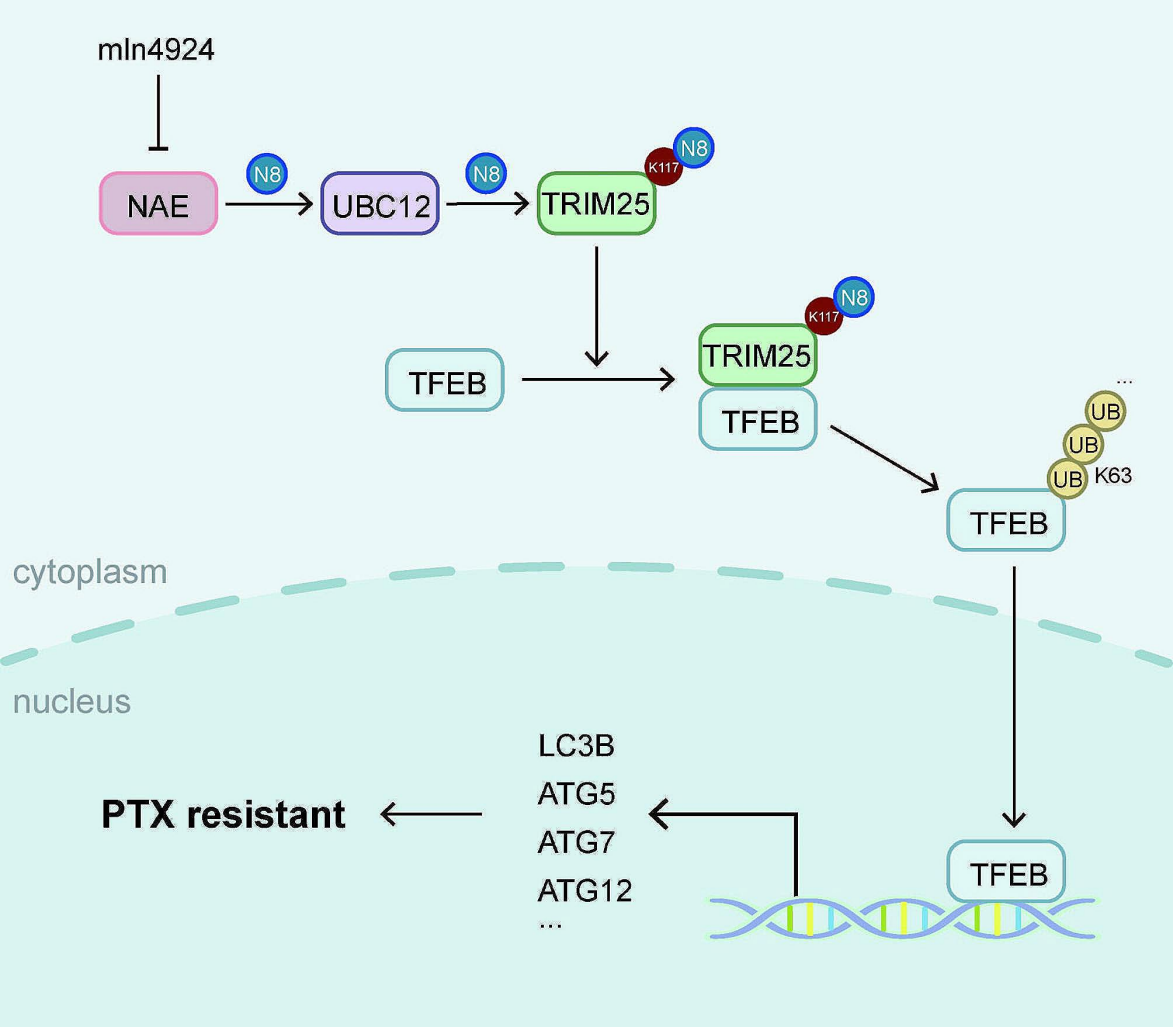
The schematic diagram of the potential mechanism in this study. N8 means NEDD8. UB means ubiquitin

Previous research has shown that NEDD8-activating enzyme (NAE) inhibitor, mln4924, enhances tumor sensitivity to oncolytic viro [39], platinum [40], and cisplatin [41] treatment through different mechanisms. Due to the lack of effective treatment for TNBC, PTX treatment resistance significantly affects patient prognosis. Here, we find that the neddylation signal is activated in PTX-insensitive TNBC, and combination therapy demonstrates a synergistic effect in cell and animal models. UBC12 mediates downstream E3 TRIM25 neddylation activation and subsequent ubiquitination substrate binding. TRIM25 is a transcriptional regulatory factor of autophagy genes, inducing TFEB nuclear translocation in a ubiquitin-dependent manner (Fig. 7).

Protein modifications serve as important post-transcriptional regulation, and their aberrations are associated with a variety of diseases. The neddylation modification introduces a 9kD protein, NEDD8, that regulates protein function in a variety of ways [13, 29, 31]. We found that importing NEDD8 caused conformational changes in TRIM25 and affected its binding to substrates. However, the results of molecular dynamics simulations are somewhat biased [42], and more evidence from structural biology is needed to reflect the real alterations. As a member of the E3 ubiquitin ligase family, TRIM25 has been reported to be associated with tumor prognosis. For example, TRIM25 activates NRF2 and promotes the progression of hepatocellular carcinoma [28], regulates the stability of EZH2, thereby promoting the resistance of colorectal cancer to oxaliplatin [43]. However, there is limited research on the upstream signals of TRIM25. It was reported that phosphorylation of tyrosine at site 278 could activate TRIM25 and regulate K63-linked polyubiquitination of RIG-I [44]. Here, we demonstrate that neddylation is also an upstream regulatory signal of TRIM25 and is associated with the progression of TNBC.

TFEB as a key autophagy regulator, influences the development of tumors [45, 46]. Small molecule inhibitors targeting TFEB have also been developed and tried for tumor therapy [47]. In addition to the extensively studied phosphorylation regulation, other post-translational modifications have been reported to influence the nucleocytoplasmic shuttling and transcriptional function of TFEB. HDAC-mediated acetylation regulates the nuclear entry of TFEB, and the acetyl group competes with lysine ubiquitination, affecting the protein stability of TFEB [48]. Alkylation of cysteine 212 affects the recognition of phosphorylated TFEB by 14-3-3 proteins, altering its cytoplasmic sequestration [24]. These modifications to TFEB regulation are independent of its phosphorylation. Interestingly, phosphorylation at different sites seems to have different regulatory effects on TFEB [8]. In this study, we found TRIM25 mediates K63-linked polyubiquitination of TFEB, in the wake of promoting its nuclear translocation and autophagic regulation.

Our findings provide a theoretical basis for the clinical combined application of neddylation inhibitors and PTX. However, in PTX-insensitive TNBC, the reasons for elevated expression of NEDD8 and UBC12, whether it is transcriptional or post-transcriptional regulation, remain unknown and worthy of further research. Additionally, given the adverse reactions exhibited by mln4924 in clinical trials of solid tumors [49], it indicates limitations of broad neddylation inhibition targeting NAE and the need for more precise targets. For example, using specific peptide inhibition of MER11 lactylation affects homologous recombination [50]. Neddylated TRIM25 presents potential as a downstream target and is worth developing.

## Conclusions

In summary, neddylation is activated in PTX-insensitive TNBC. By altering the protein conformation of TRIM25, neddylation enhances its binding with macromolecular substrates. Activated TRIM25 promotes TFEB K63-linked ubiquitination and nuclear translocation. Autophagy gene transcriptional activation mediated by the UBC12/TRIM25/TFEB axis reduces TNBC sensitivity to PTX.

### Electronic supplementary material

Below is the link to the electronic supplementary material.

Supplementary Material 1

Supplementary Material 2

Supplementary Material 3

## Data Availability

All data and materials are available upon request by contacting the corresponding author.

## Abbreviations

NEDD8,: neural precursor cell expressed developmentally downregulated protein 8
TNBC: triple-negative breast cancer
PTX: paclitaxel
NAE: NEDD8-activating enzyme
TRIM25: tripartite motif containing 25
TFEB: transcription factor EB
LPS: lipopolysaccharide
PTM: post-translational modification
IHC: Immunohistochemistry
MDC: Monodansyl cadaverine
