# Supplementary material for: Neddylation activated TRIM25 desensitizes triple-negative breast cancer to paclitaxel via TFEB-mediated autophagy
Source: J Exp Clin Cancer Res. 2024 Jun 26;43:177. doi: 10.1186/s13046-024-03085-w (PMC11201311; doi:10.1186/s13046-024-03085-w)
Supplement: Supplementary file 1 — Supplementary Material 1 [file 13046_2024_3085_MOESM1_ESM.docx]

**
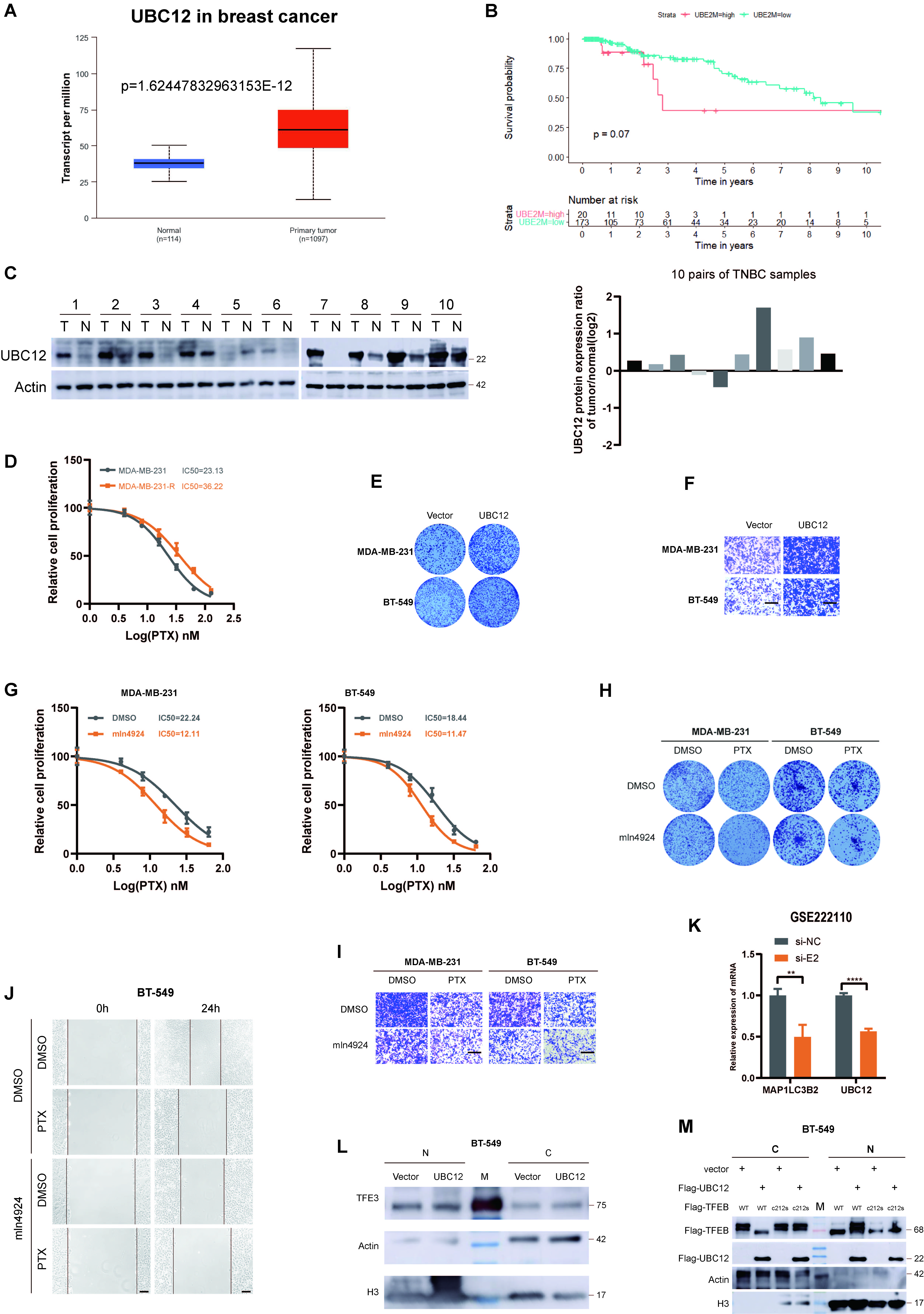
**

**Fig.S1 A** UBC12 mRNA expression in breast cancer and normal tissue based on UALCAN database (https://ualcan.path.uab.edu/). **B** UBC12 expression correlated with survival in TCGA database. **C** Western blot analysis of UBC12 protein in breast cancer and normal tissue. (T means tumor tissue. N means normal tissue.) **D** MDA-MB-231 and MDA-MB-231-R cells were treated with a serial dose of PTX for 24 hours and subjected to MTT assay. The IC50 values of PTX in each group were indicated. **E-F** Colony formation and transwell migration assays of two cells when UBC12 was overexpression. Scale bars, 200 µm. **G** TNBC cells were treated with a serial dose of PTX and a fixed dose of mln4924 (100nM) for 24 hours and subjected to MTT assay. The IC50 values of PTX in each group are indicated. **H-J** Colony formation, wound healing and transwell migration assays after drug treatment. Scale bars, 200 µm. **K** Statistical analysis of transcriptome sequencing dataset GSE222110. **L** Western blot analysis of TFE3 protein in nucleus and cytoplasm. **M** Western blot analysis of the expression level of TFEB in nucleus and cytoplasm when UBC12 was overexpressed, with or without TFEB C212S mutant. Data are presented as mean ± SEM. ***p < 0.05, **p < 0.01, ***p < 0.001, ****p < 0.0001**


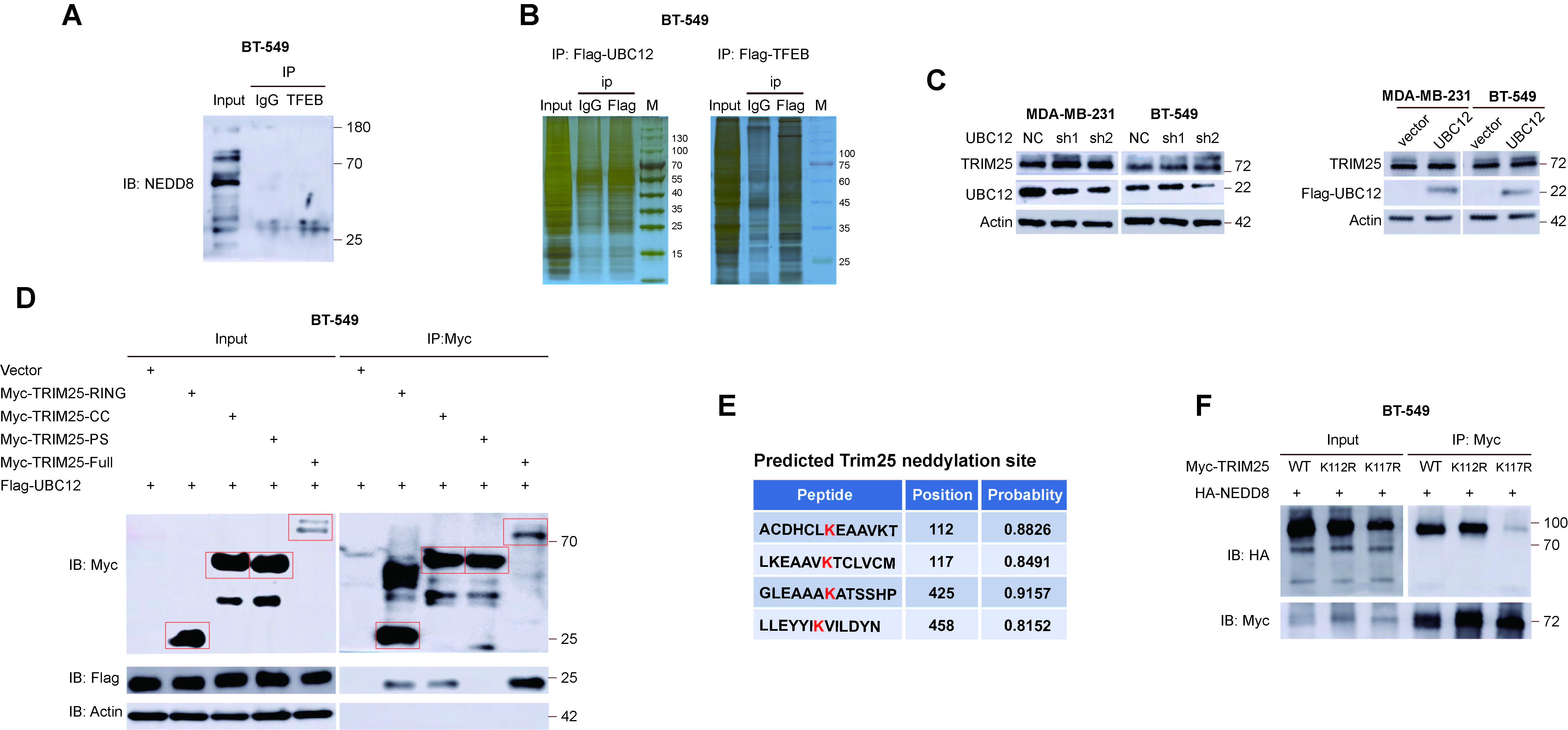


**Fig.S2 A** Western blot analysis of TFEB neddylation in BT-549 cell. **B** Silver staining of proteins pulled down by IP experiments. **C** Western blot analysis of TRIM25 and UBC12 when UBC12 was overexpression. **D** Western blot analysis of UBC12 and segmented TRIM25 in BT-549 cell. **E** Predicted high-confidence neddylation sites in TRIM25. **F** Western blot analysis of neddylation level of TRIM25 in BT-549 cell when transfected with TRIM25 wild-type or mutant plasmids.

**
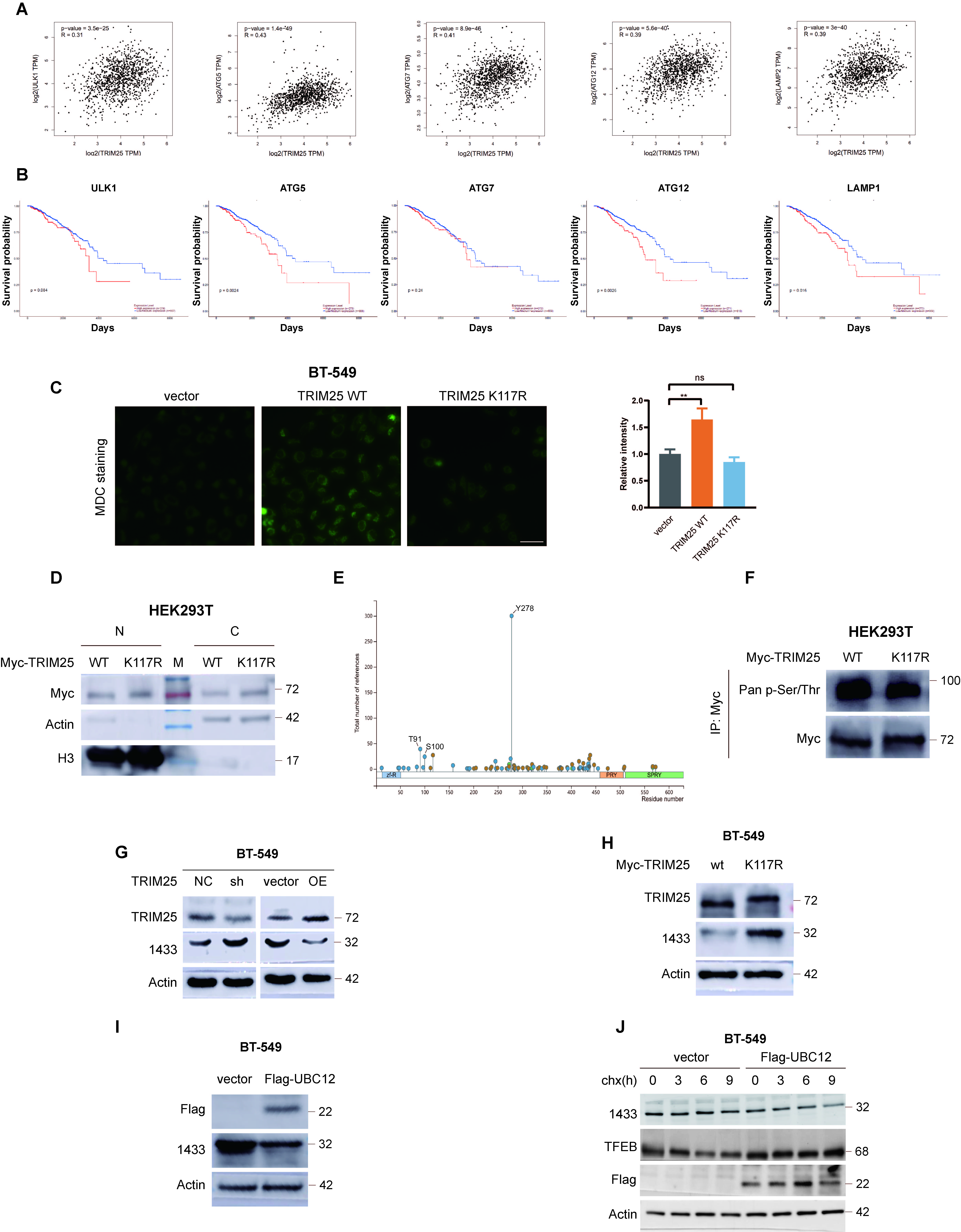
**

**Fig.S3 A** Correlation analysis of the expression of TRIM25 and autophagy related genes in breast cancer tissues from the TCGA database. (http://gepia.cancer-pku.cn/) **B** Autophagy related genes expression correlated with survival of BC patients in TCGA database. (https://ualcan.path.uab.edu/) **C** MDC staining assays of acidic vesicular organelles formation when transfected with TRIM25 wild-type or mutant plasmids. Scale bars, 50 µm. **D** Western blot analysis of TRIM25 protein in nucleus and cytoplasm. **E** Prediction of TRIM25 modification sites using the website Phosphosite (www.phosphosite.org). Blue dots represent amino acids capable of undergoing phosphorylation modification and yellow dots represent lysine. **F** Western blot analysis of phosphorylation levels of serine and threonine in TRIM25. **G-J** Western blot analysis of UBC12, TRIM25, 14-3-3 proteins in whole BT-549 cell lysate after plasmid transfection or CHX treatment. Data are presented as mean ± SEM. ***p < 0.05, **p < 0.01, ***p < 0.001, ****p < 0.0001**
