# Supplementary material for: Neddylation activated TRIM25 desensitizes triple-negative breast cancer to paclitaxel via TFEB-mediated autophagy
Source: J Exp Clin Cancer Res. 2024 Jun 26;43:177. doi: 10.1186/s13046-024-03085-w (PMC11201311; doi:10.1186/s13046-024-03085-w)
Supplement: Supplementary file 2 — Supplementary Material 2 [file 13046_2024_3085_MOESM2_ESM.docx]

**Supplemental Tables 1**

**Primers used in this research**

| **Gene** | **Sequences(5’-3’)** |
| --- | --- |
| h-TFEB-F | CCTGGAGATGACCAACAAGCAG |
| h-TFEB-R | TAGGCAGCTCCTGCTTCACCAC |
| h-UBC12-F | AGCCAGTCCTTACGATAAACTCC |
| h-UBC12-R | TGCACGTTCTGCTCAAACAGCC |
| h-TRIM25-F | AAAGCCACCAGCTCACATCCGA |
| h-TRIM25-R | GCGGTGTTGTAGTCCAGGATGA |
| h-LC3B-F | GAGAAGCAGCTTCCTGTTCTGG |
| h-LC3B-R | GTGTCCGTTCACCAACAGGAAG |
| h-ATG5-F | GCAGATGGACAGTTGCACACAC |
| h-ATG5-R | GAGGTGTTTCCAACATTGGCTCA |
| h-beta-actin-F | CACCATTGGCAATGAGCGGTTC |
| h-beta-actin-R | AGGTCTTTGCGGATGTCCACGT |

**Antibodies used in this research**

| **Protein** | **cat** | **company** |
| --- | --- | --- |
| Anti-TFEB | A21928 | abclonal |
| Anti-pan-1433 | A23829 | abclonal |
| Anti-UBC12 | 14520-1-AP | proteintech |
| Anti-p62 | T55546 | abmart |
| Anti-LC3B | E5Q2K | CST |
| Anti-p-UB | 62802S | CST |
| Anti-TRIM25 | A12938 | abclonal |
| Anti-NEDD8 | A22568 | abclonal |
| Anti-pan-p-ser/thr | T91067 | abmart |
| Anti-Myc-tag | AE010 | abclonal |
| Anti-Flag-tag (M) | 66008-4-Ig | proteintech |
| Anti-Flag-tag (R) | 20543-1-AP | proteintech |
| Anti-HA-tag | AE008 | abclonal |
| Anti-Control IgG (M) | AC011 | abclonal |
| Anti-Control IgG (R) | AC005 | abclonal |
| Anti-GAPDH | HRP-60004 | proteintech |
| Anti-Histone H3 | A2348 | abclonal |
| Anti-beta-actin | HRP-60008 | proteintech |
| HRP Goat Anti-Rabbit IgG | AS014 | abclonal |
|  |  |  |
| HRP Goat Anti-Mouse IgG | AS003 | abclonal |
| Avoidance Antibody (H+L) Secondary Antibody | M21008 | abmart |
|  |  |  |
