# Supplementary material for: Neddylation activated TRIM25 desensitizes triple-negative breast cancer to paclitaxel via TFEB-mediated autophagy
Source: J Exp Clin Cancer Res. 2024 Jun 26;43:177. doi: 10.1186/s13046-024-03085-w (PMC11201311; doi:10.1186/s13046-024-03085-w)

Marker we used for western bolt

26616

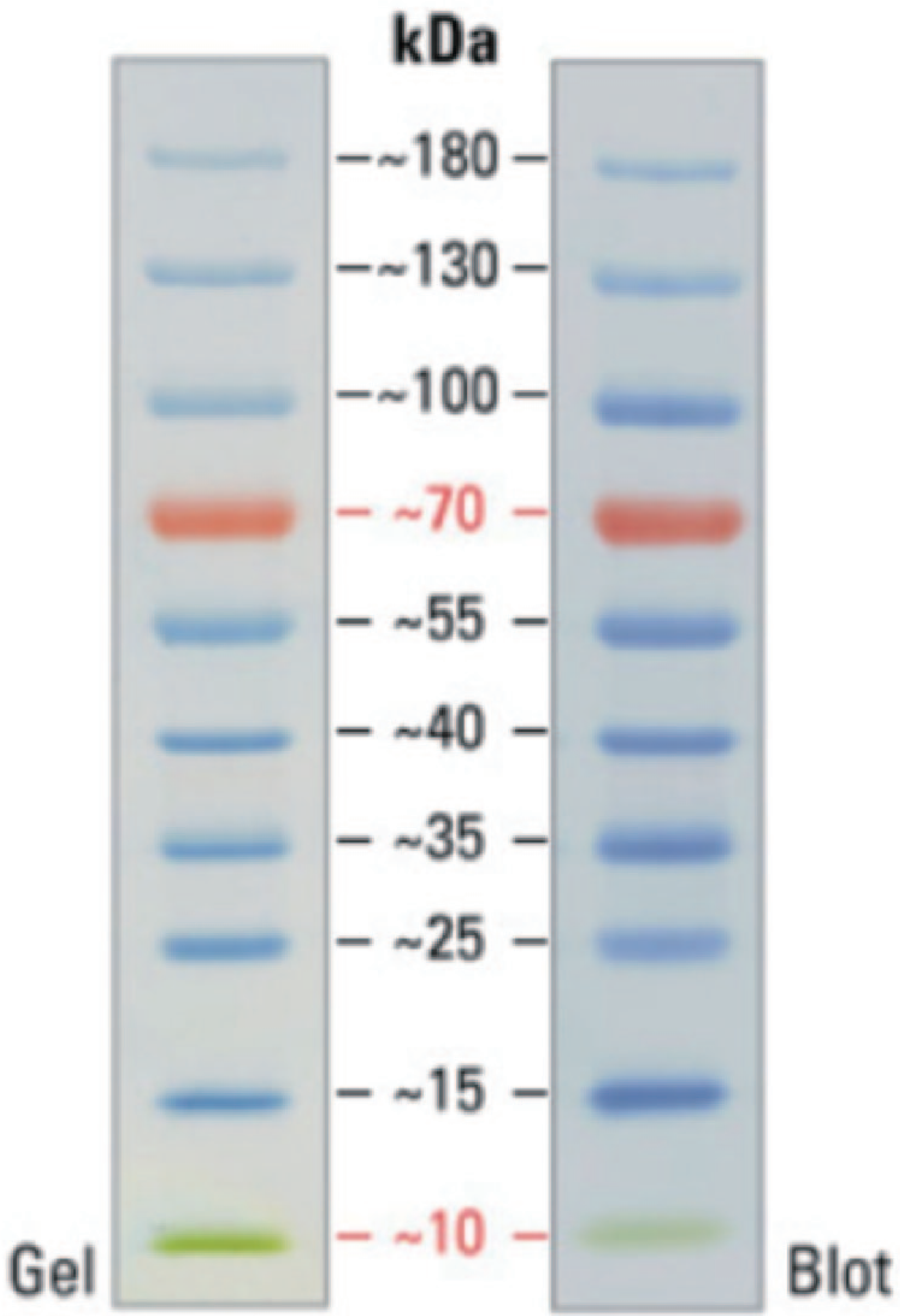

Abclonal RM19001

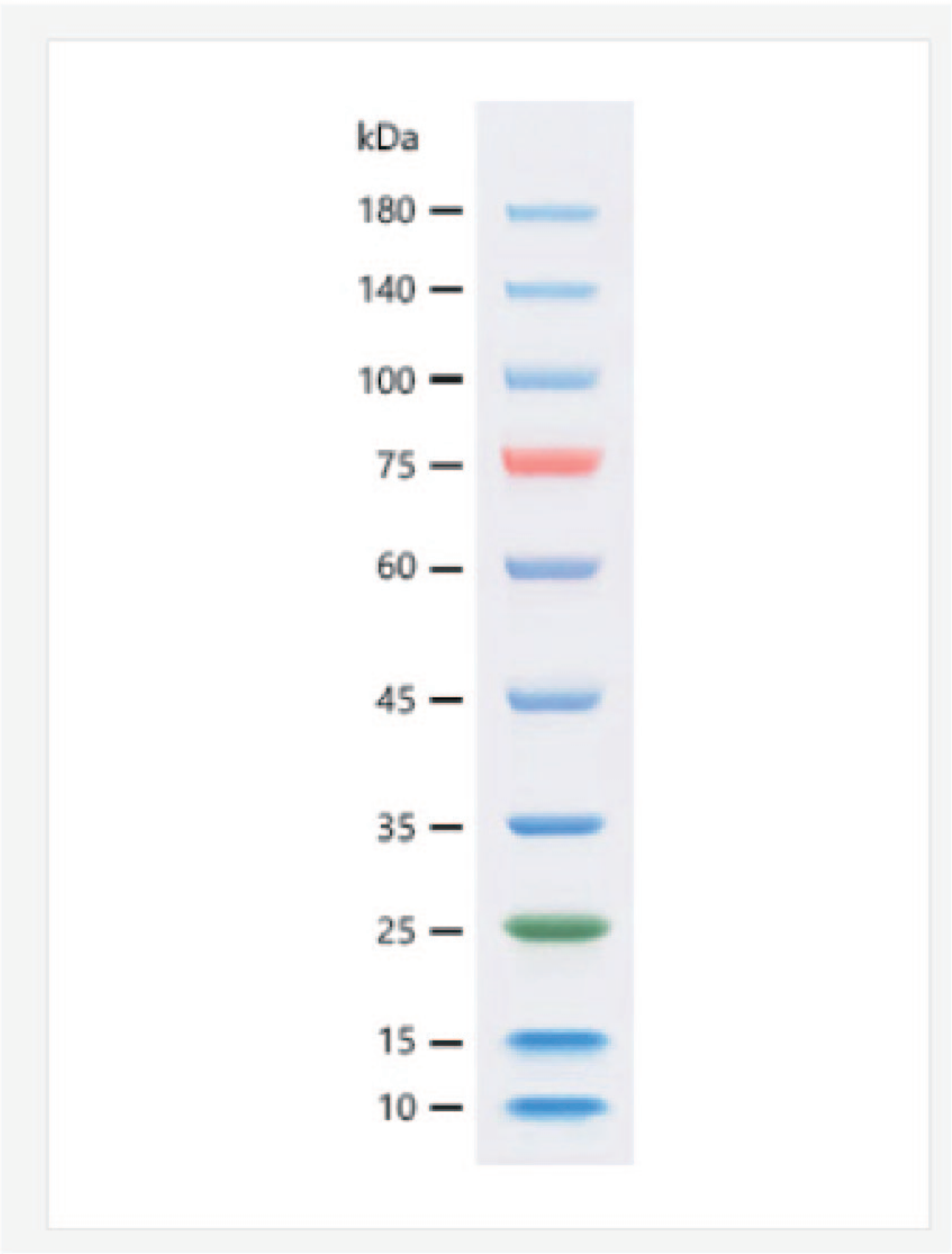

Figure2

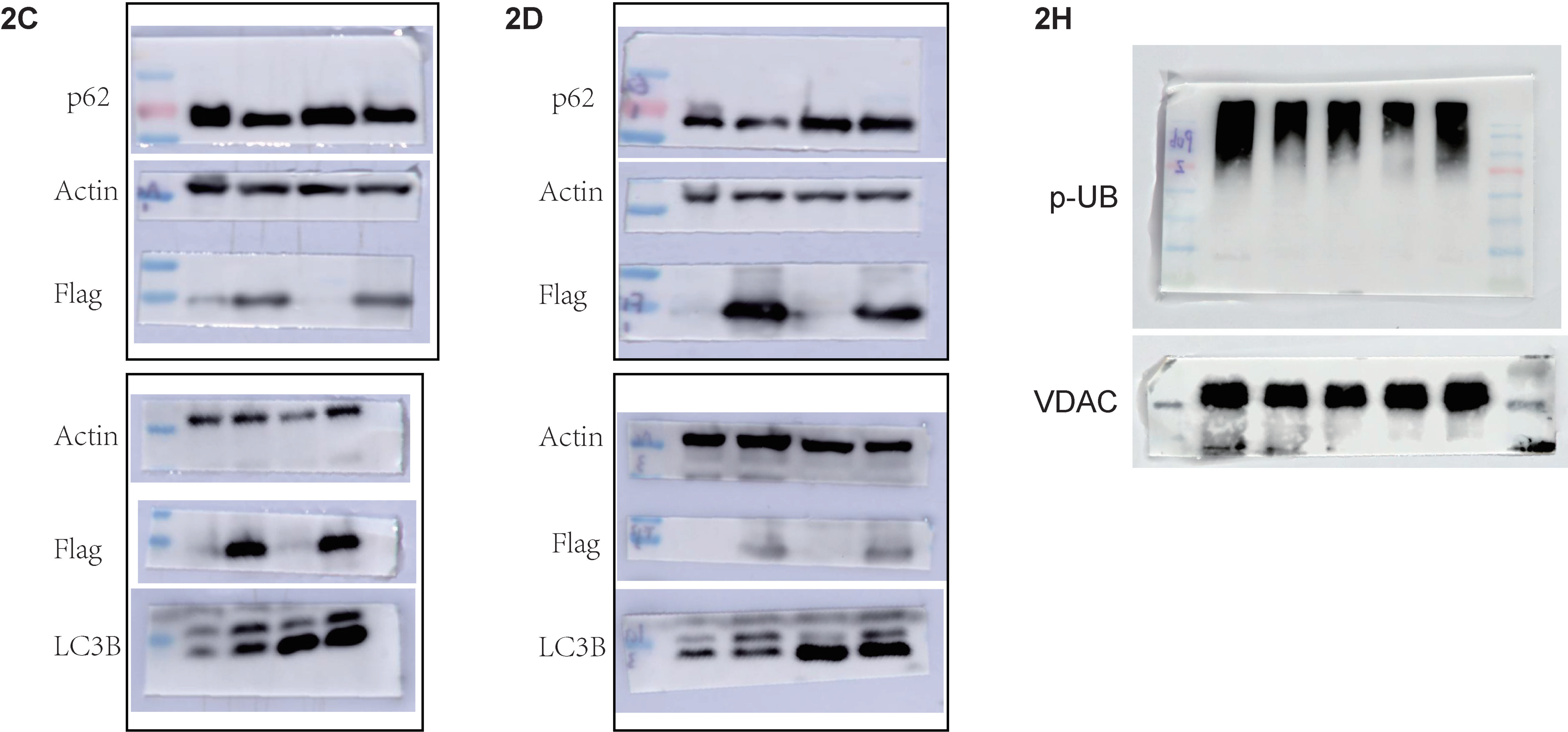

2C
p62
Actin
Flag
Actin
Flag
LC3B
2D
p62
Actin
Flag
Actin
Flag
LC3B
2H
p-UB
VDAC

Figure3

3A

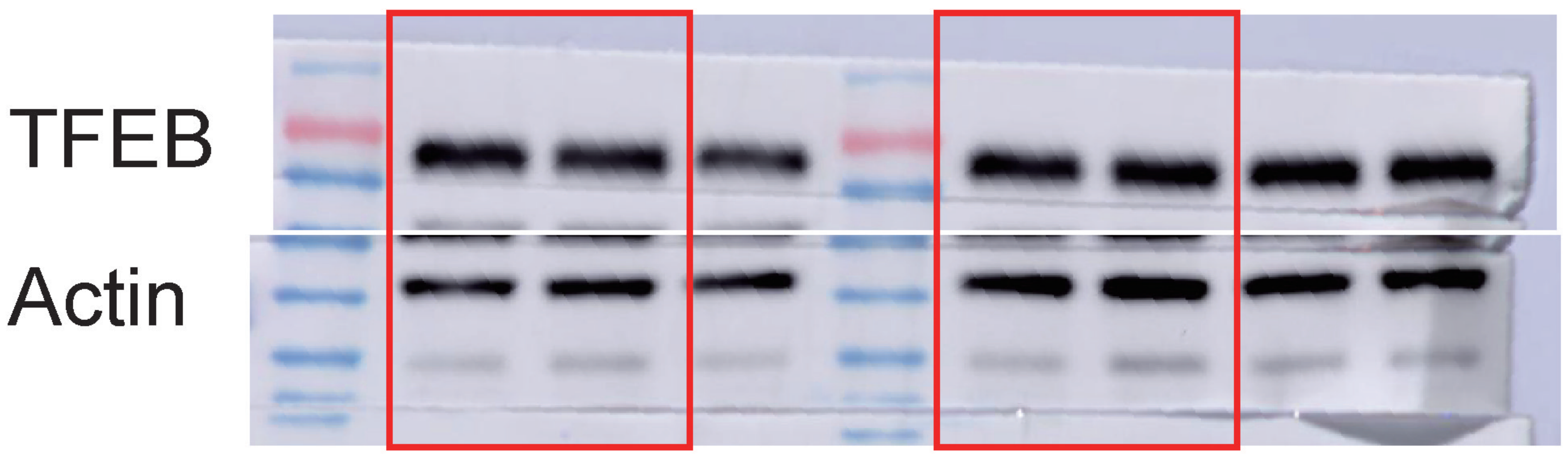

3B

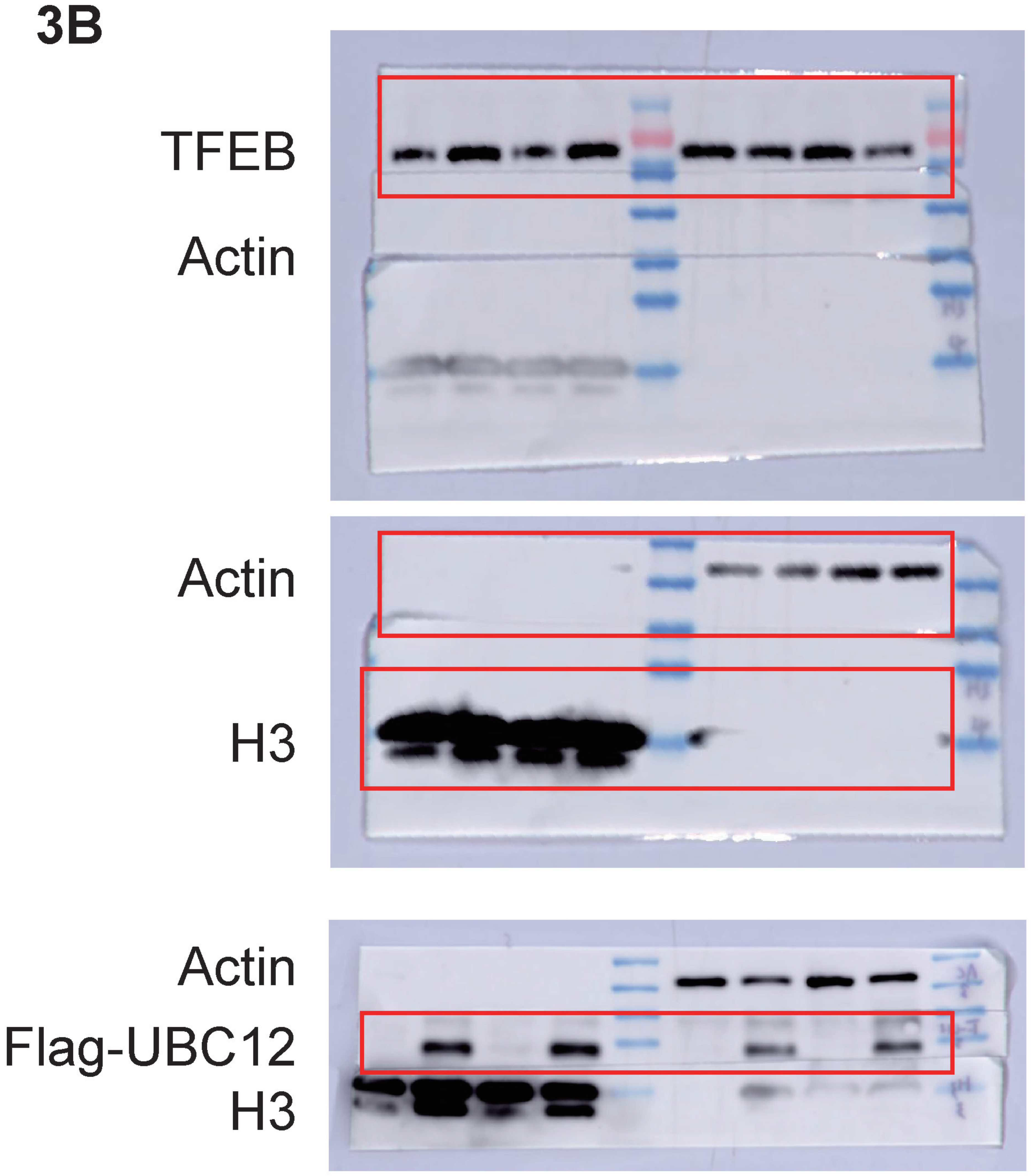

3E

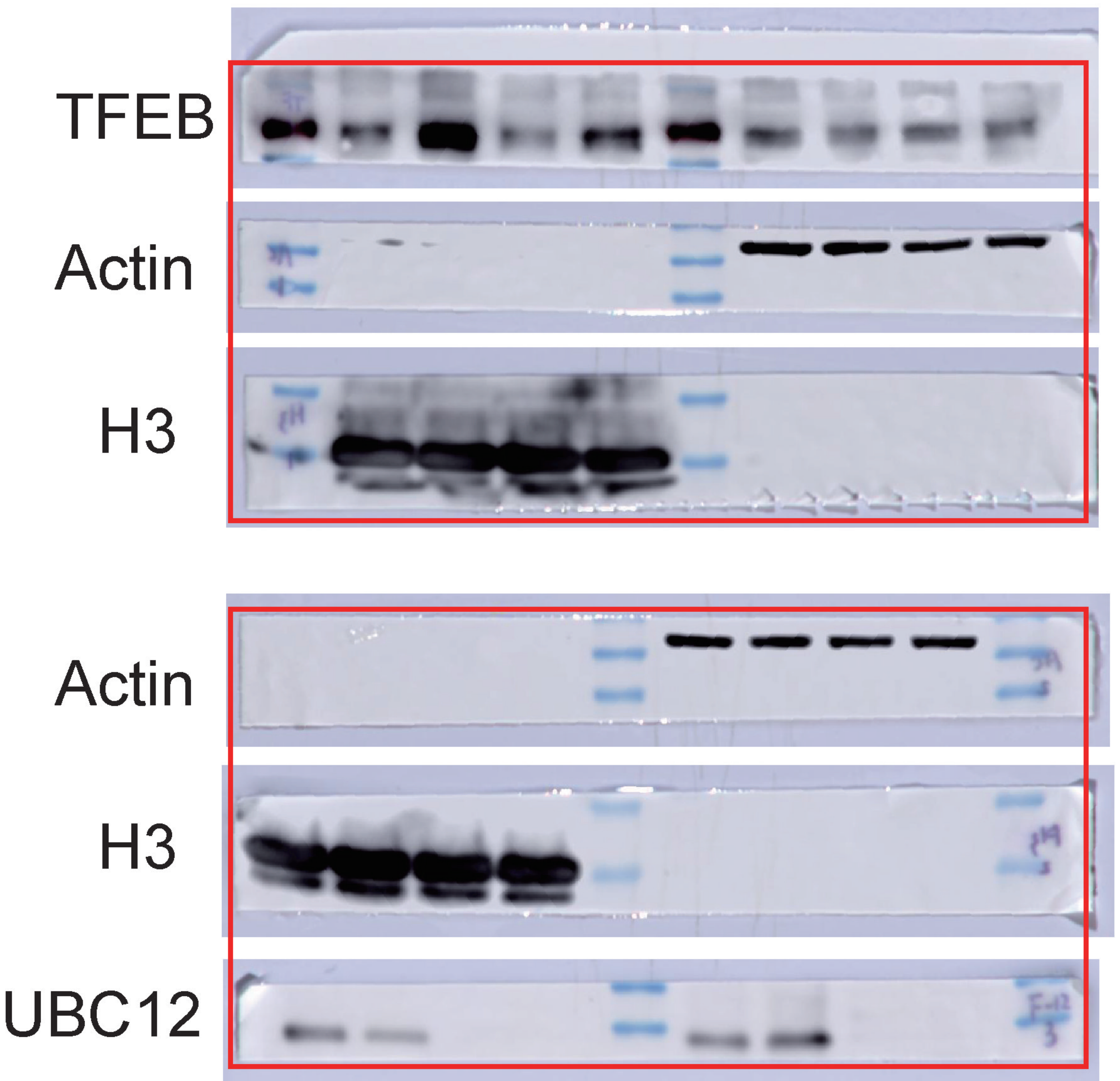

3F

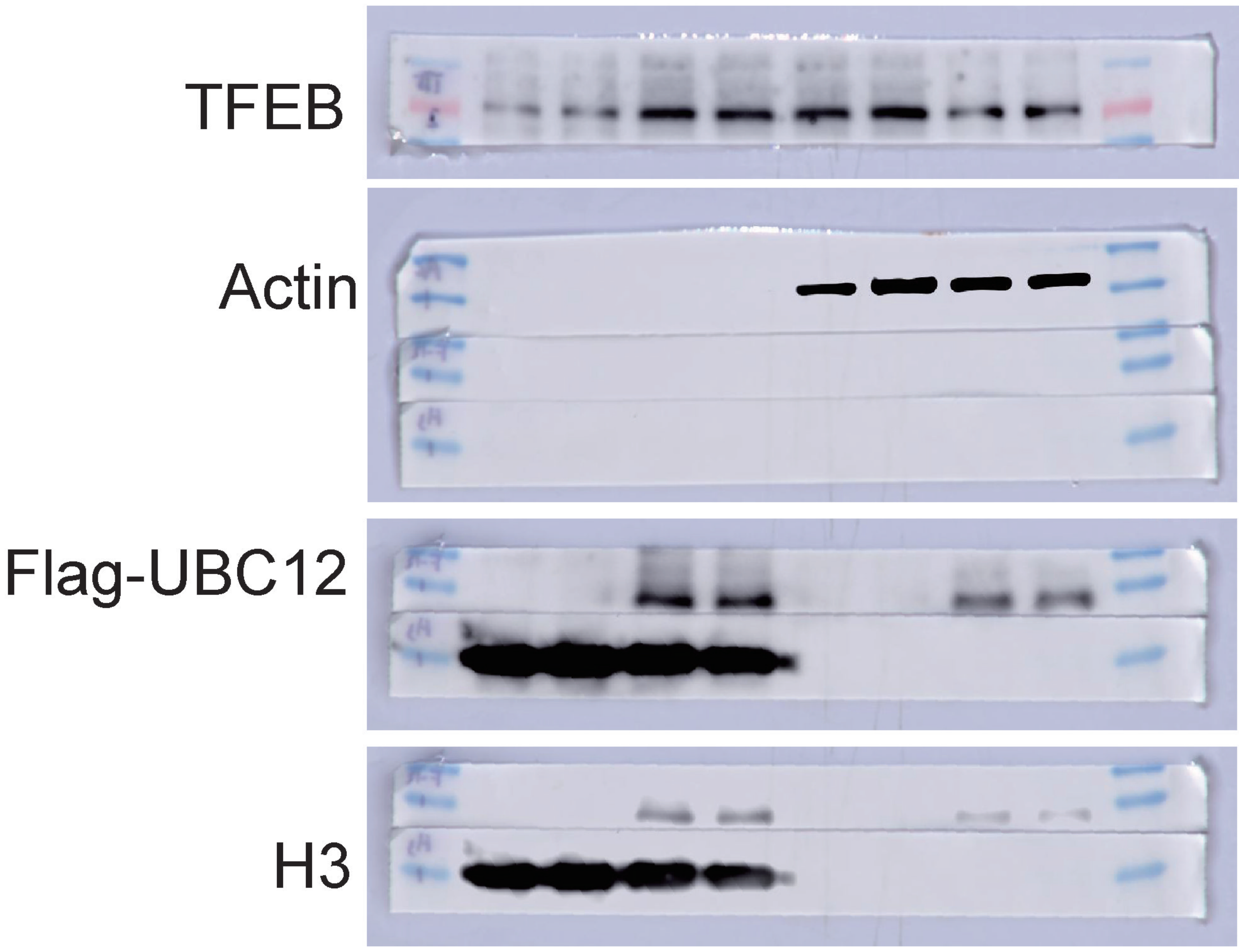

Figure4

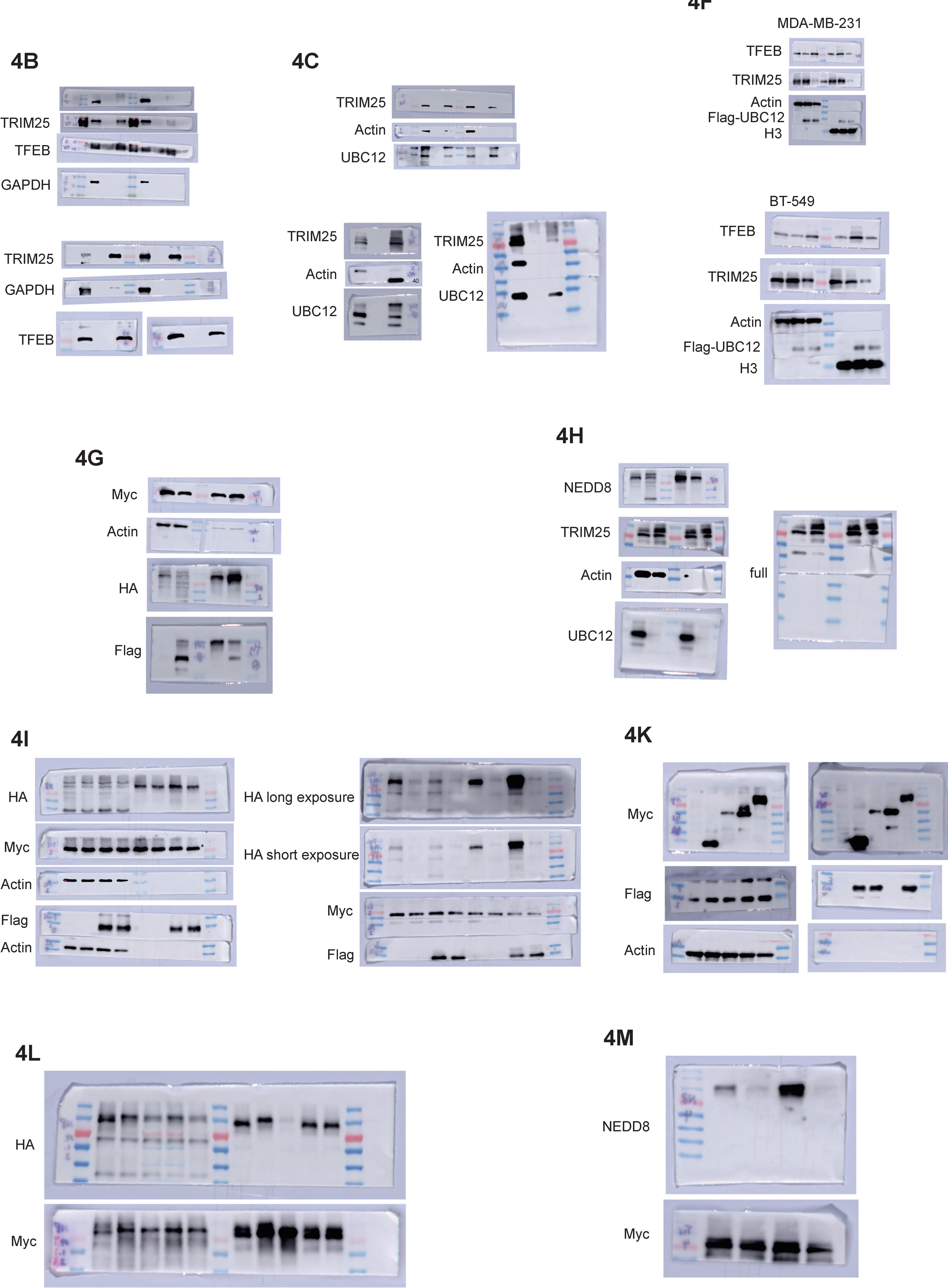

4B
TRIM25
TFEB
GAPDH
TRIM25
GAPDH
TFEB
4C
TRIM25
Actin
UBC12
TRIM25
Actin
UBC12
TRIM25
Actin
UBC12
4F
MDA-MB-231
TFEB
TRIM25
Actin
Flag-UBC12
H3
BT-549
TFEB
TRIM25
Actin
Flag-UBC12
H3
4G
Myc
Actin
HA
Flag
4H
NEDD8
TRIM25
Actin
UBC12
full
4I
HA
Myc
Actin
Flag
Actin
HA long exposure
HA short exposure
Myc
Flag
4K
Myc
Flag
Actin
4L
HA
Myc
4M
NEDD8
Myc

Figure5

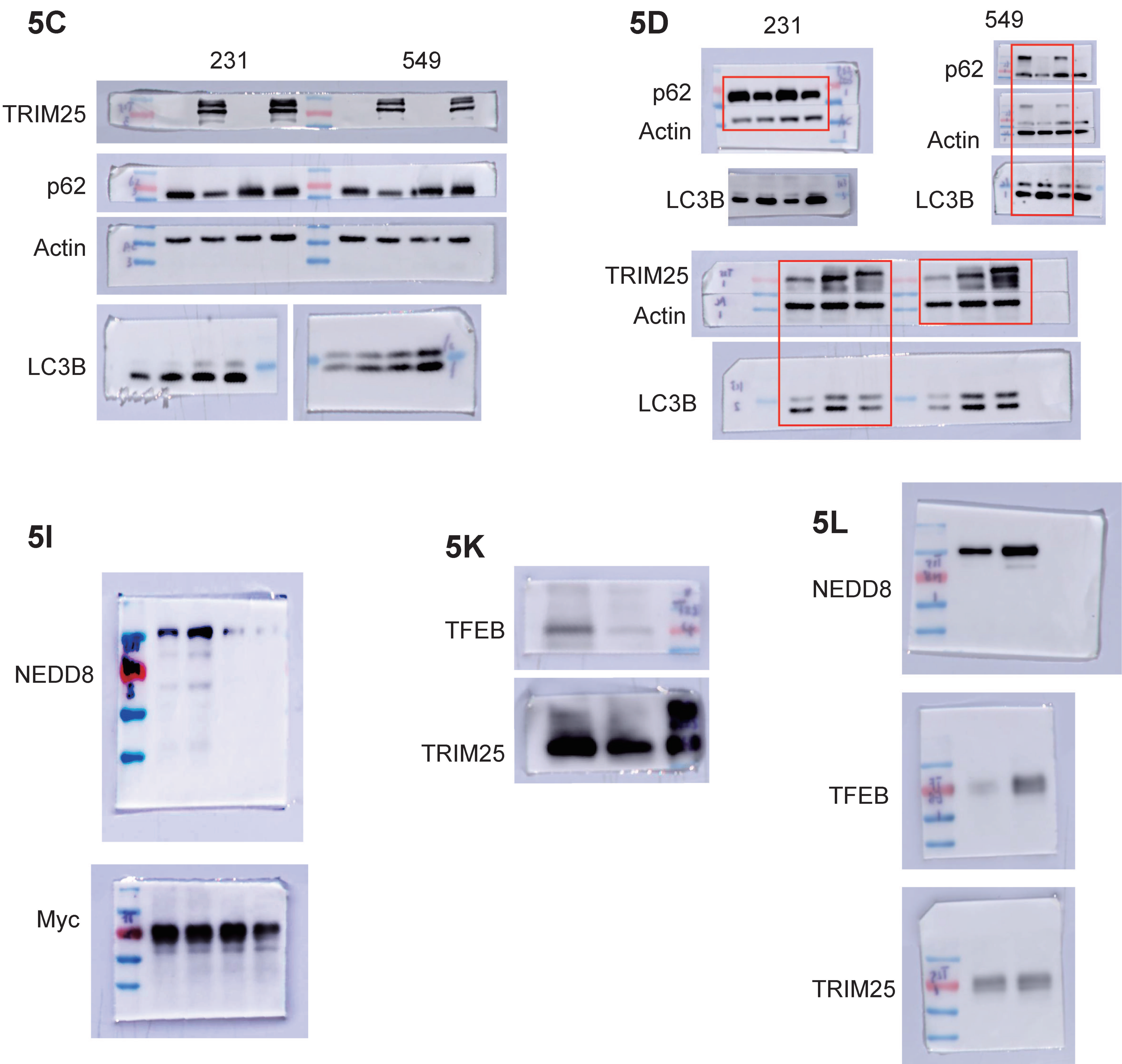
5C
231
549
TRIM25
p62
Actin
LC3B
5D
231
549
p62
Actin
LC3B
TRIM25
Actin
LC3B
5I
NEDD8
Myc
5K
TFEB
TRIM25
5L
NEDD8
TFEB
TRIM25

Figure6

6A

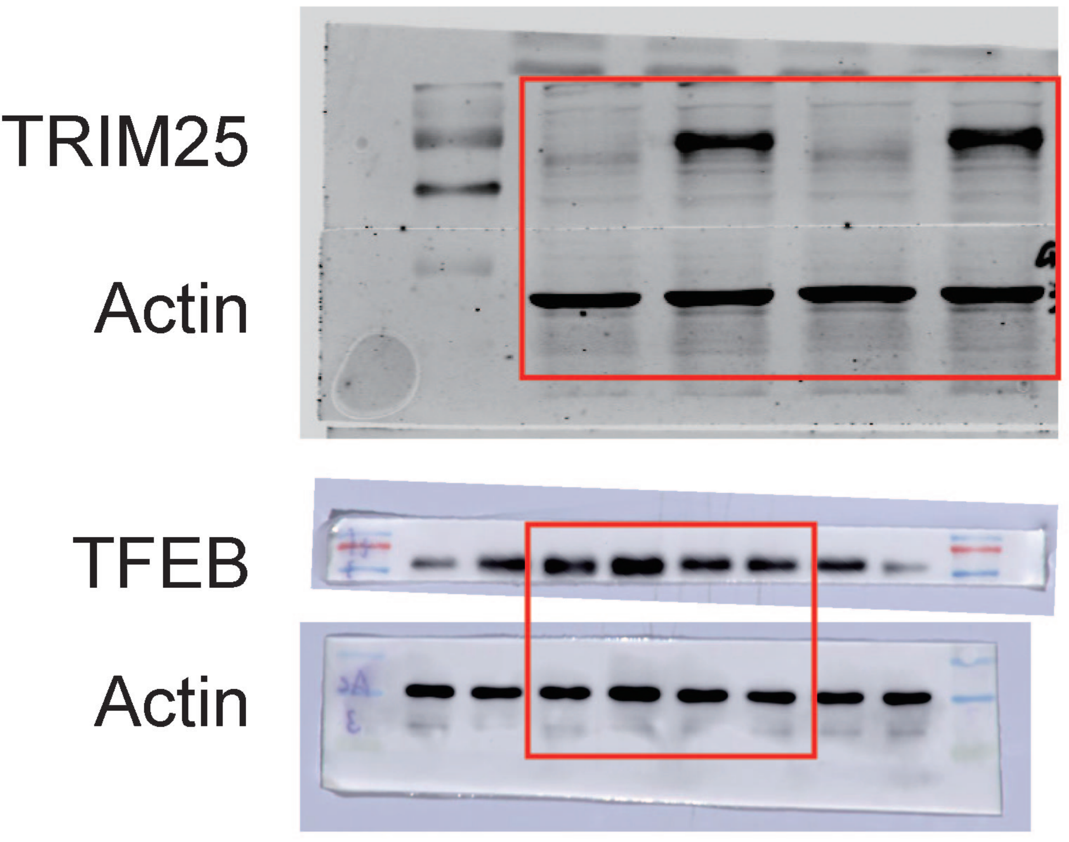

6B

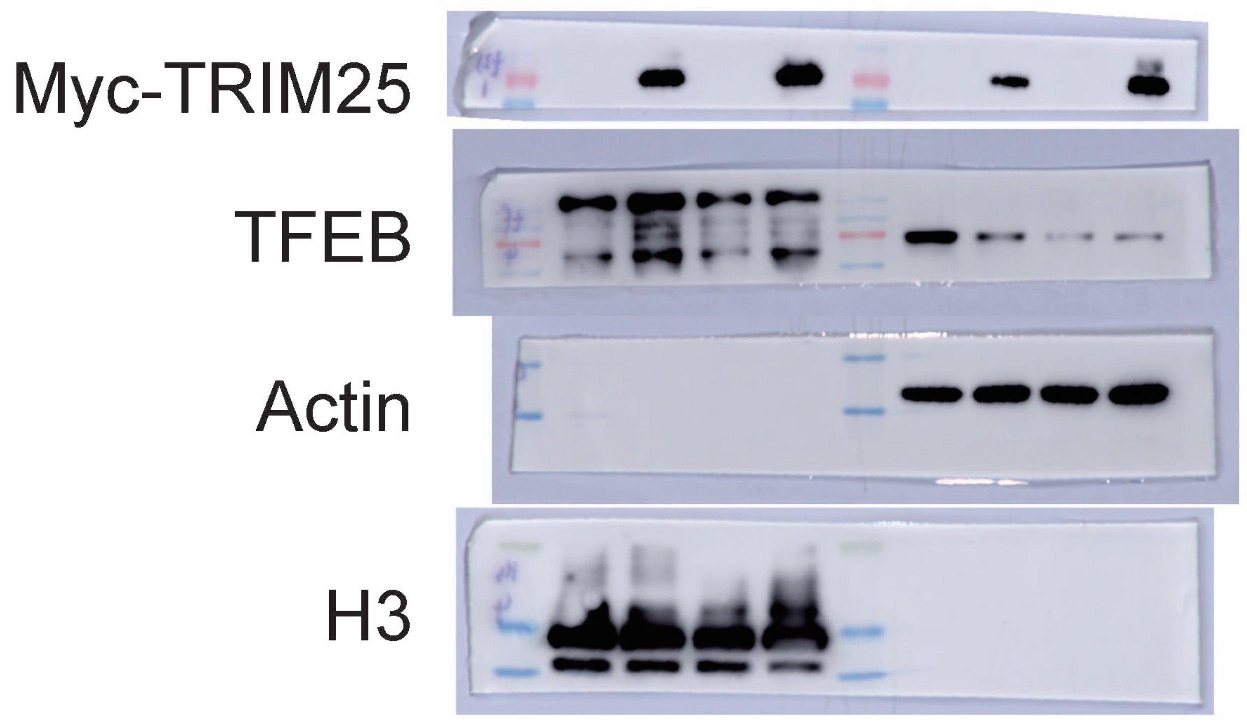

6D

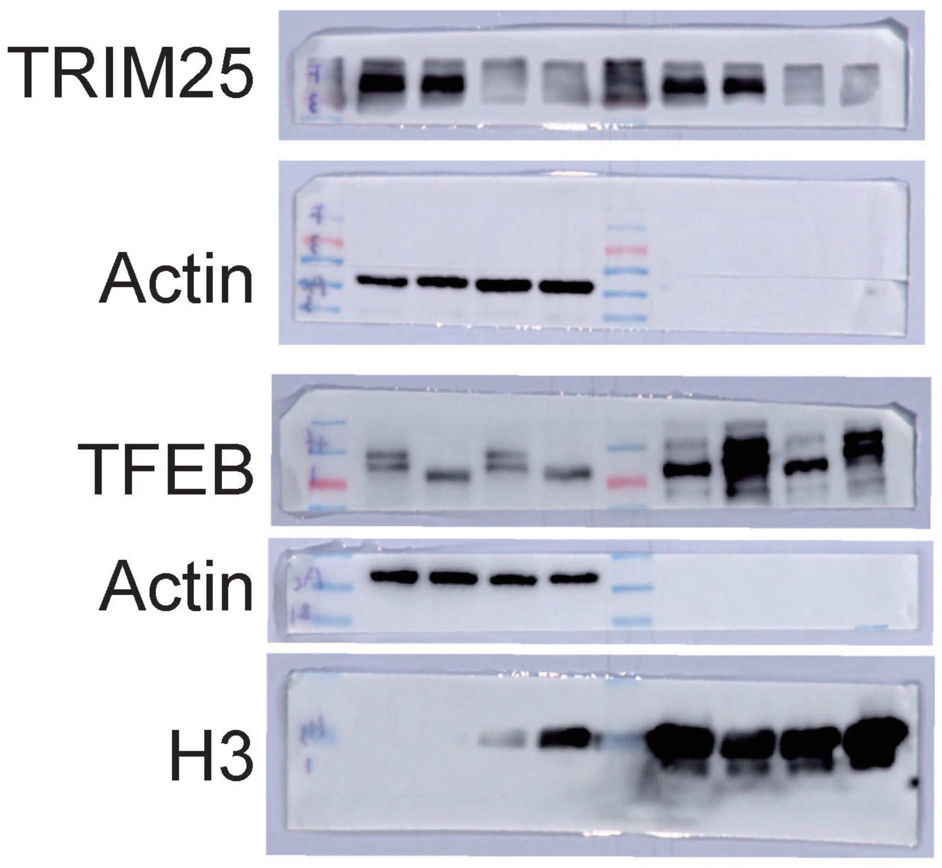

6E

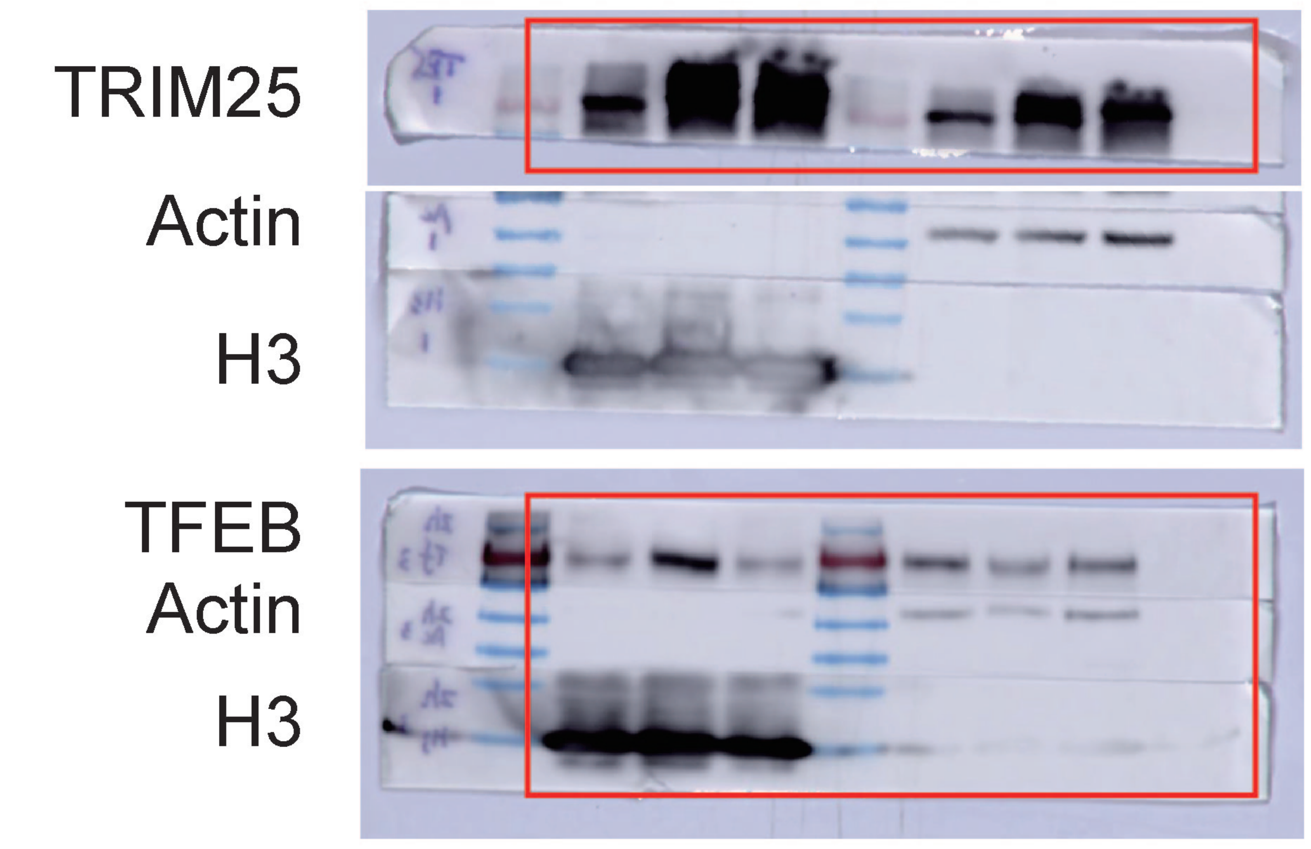

6F

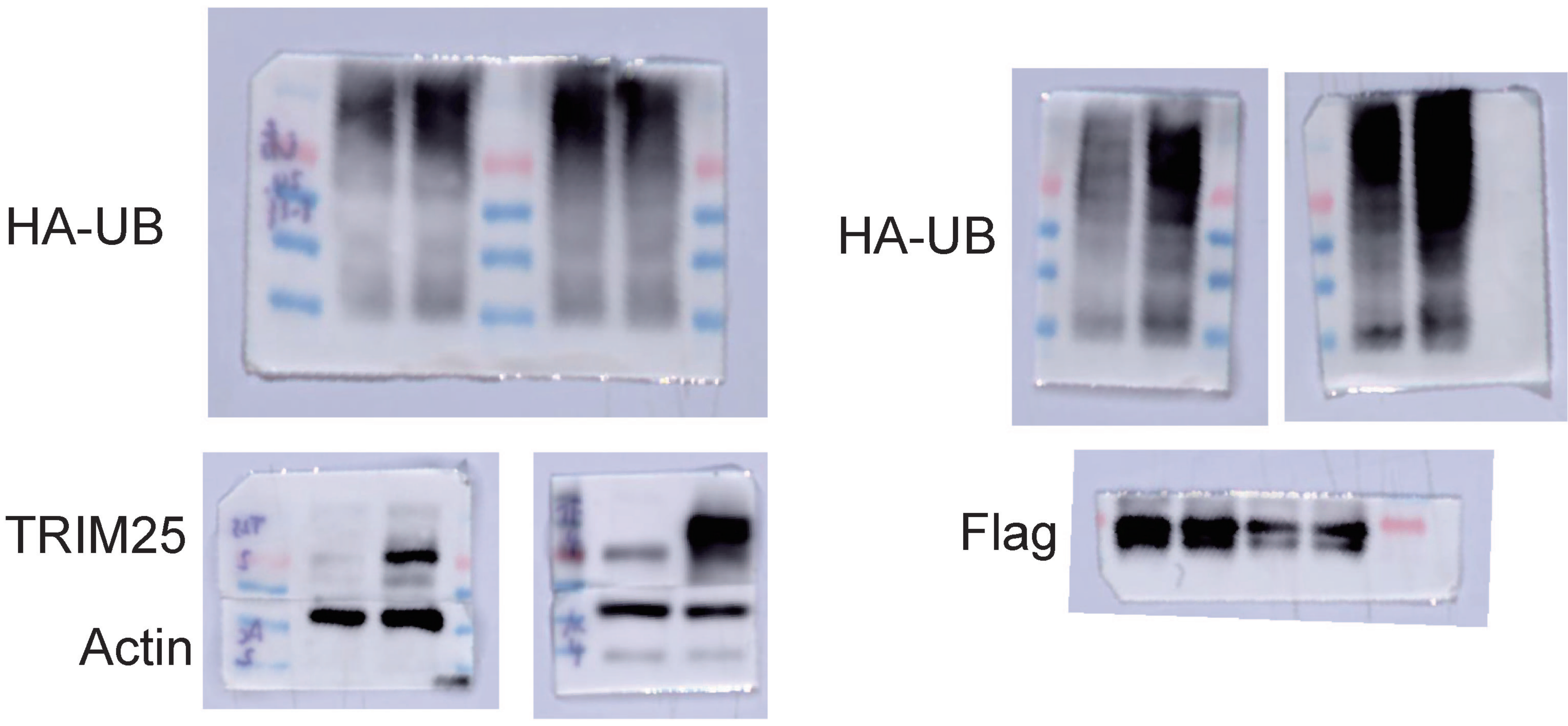

6G and H

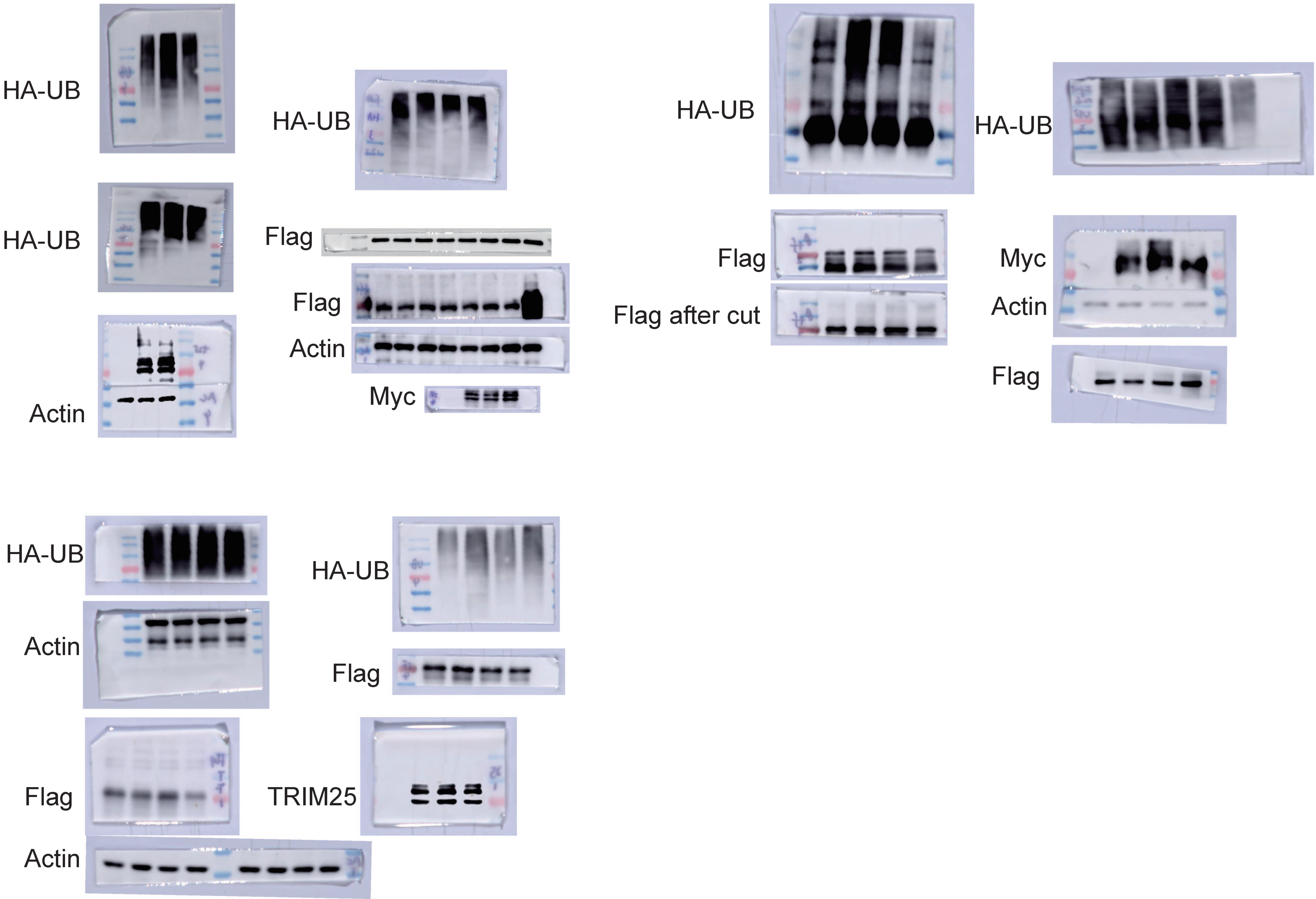

FigureS1

S1C

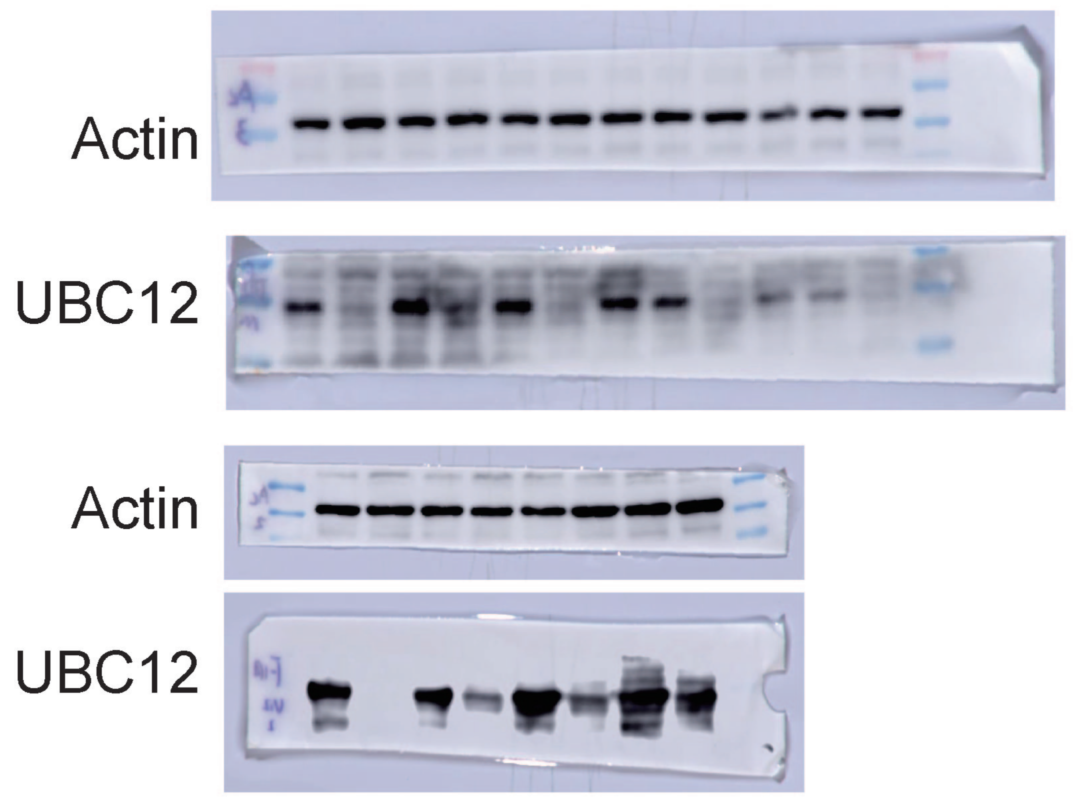

S1L

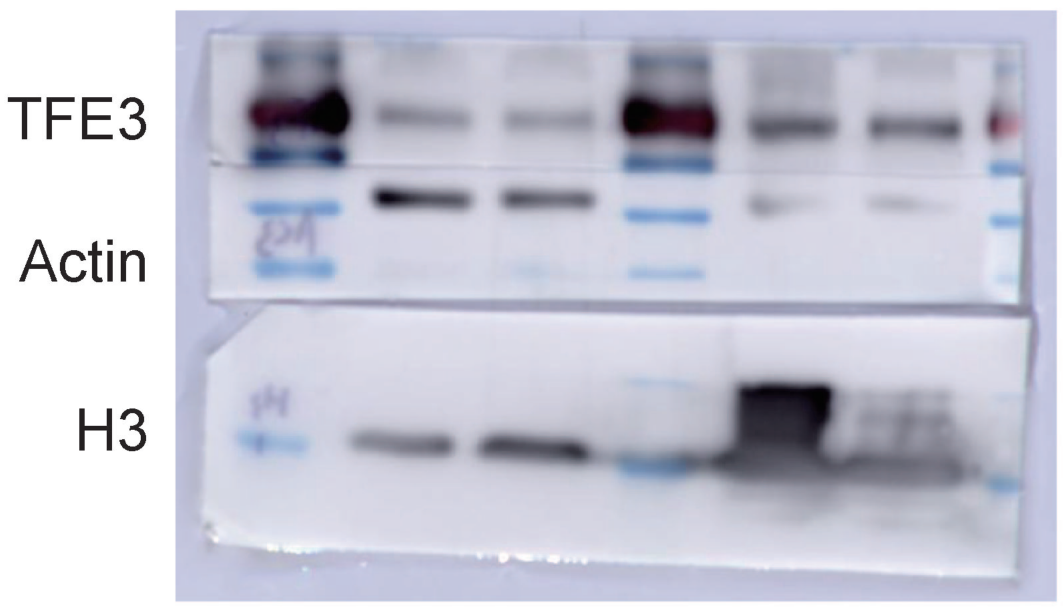

S1M

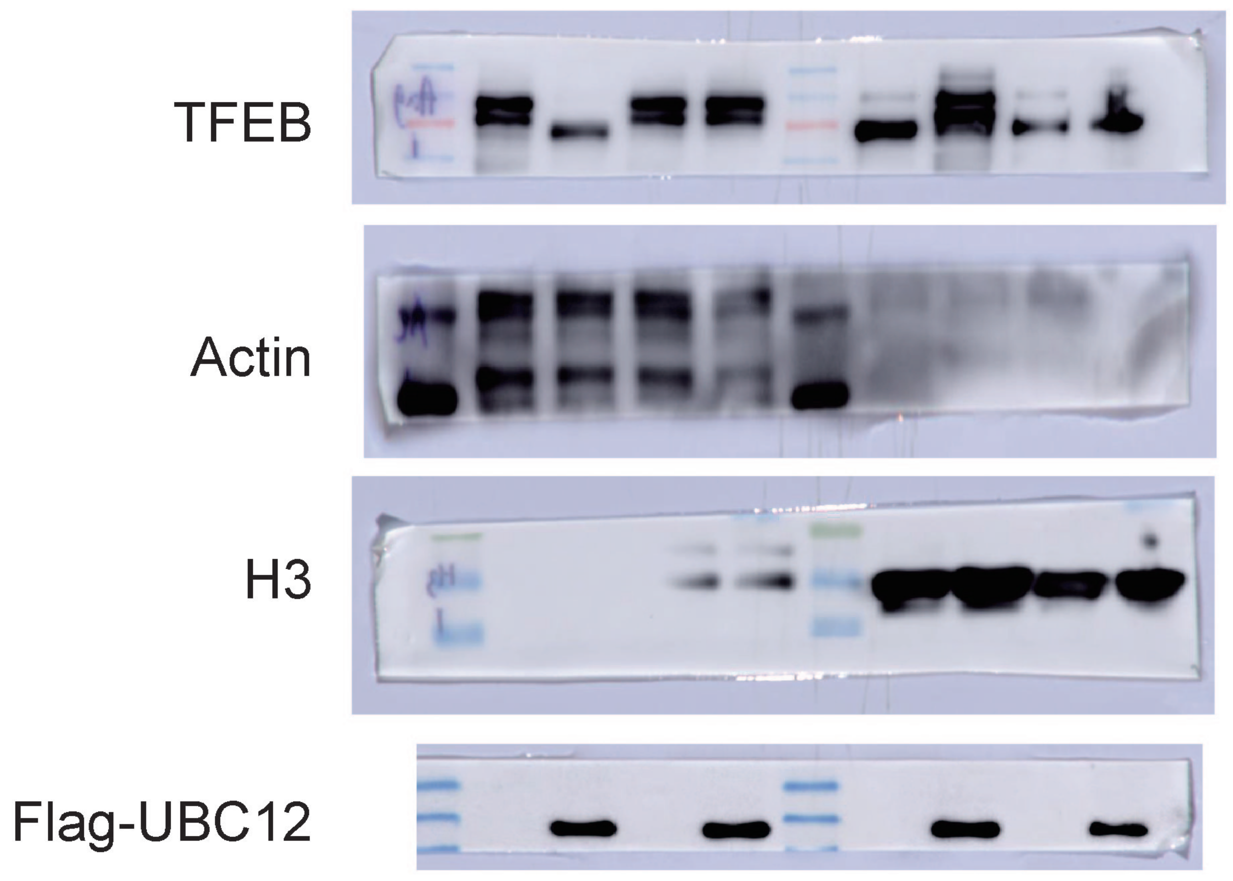

FigureS2

S2A

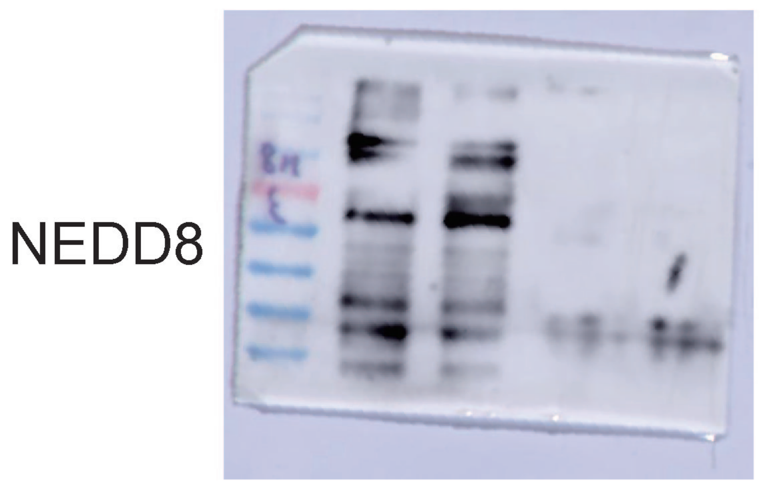

S2C

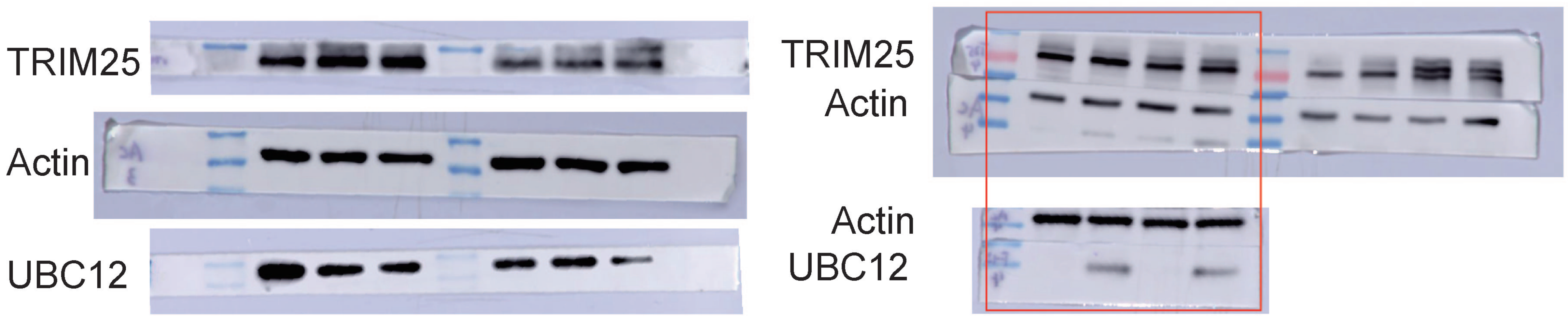

S2D

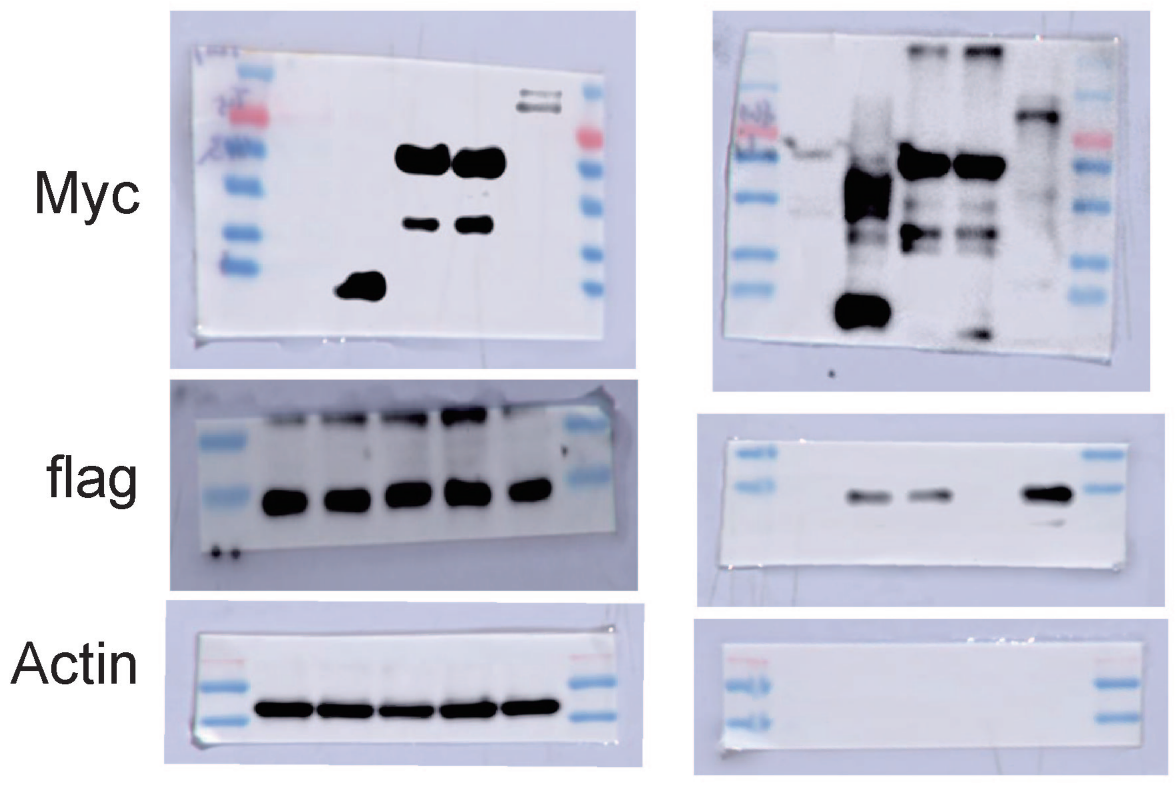

S2F

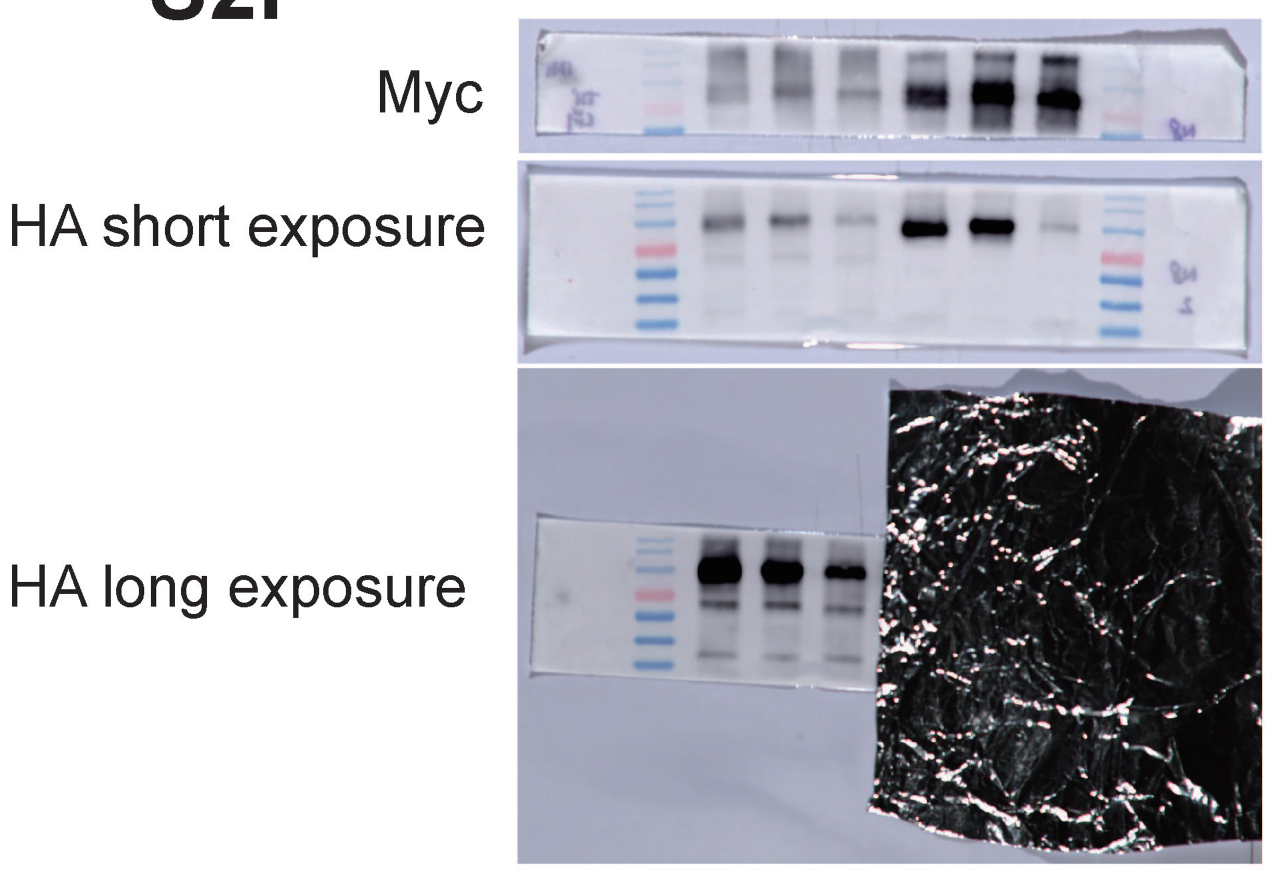

FigureS3

S3D

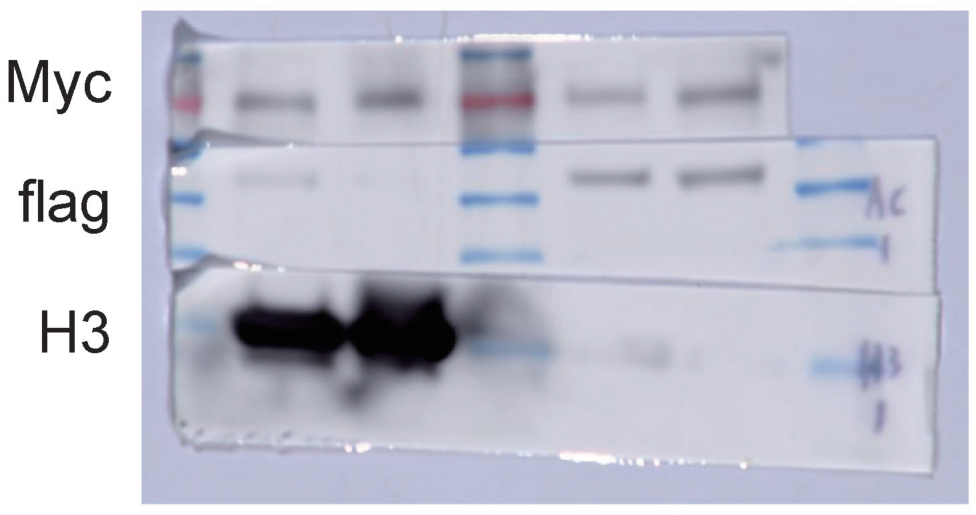

S3F

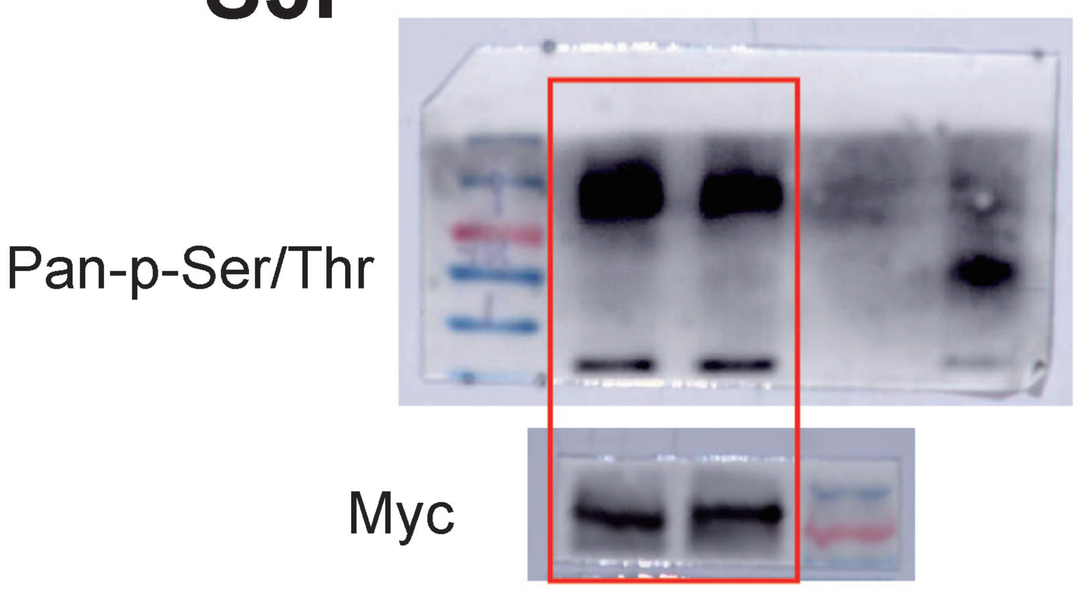

S3G

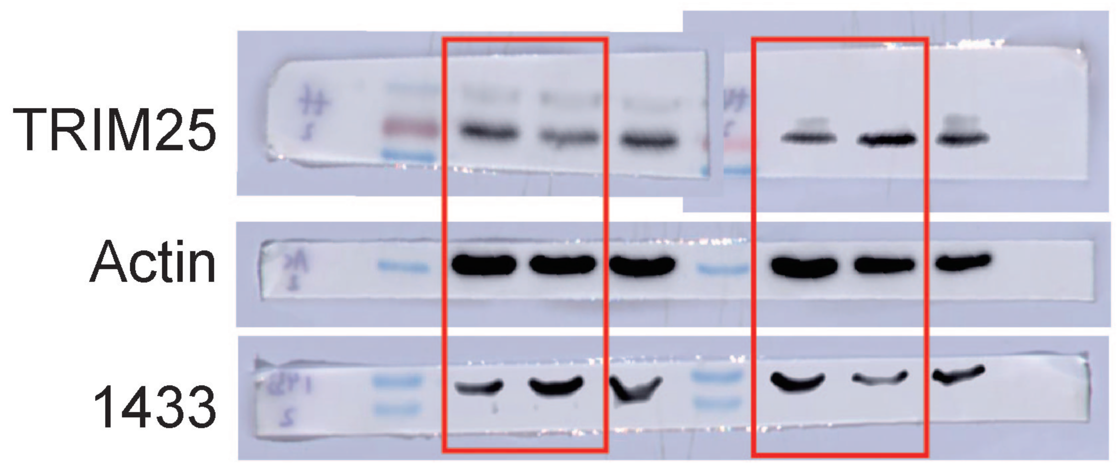

S3H

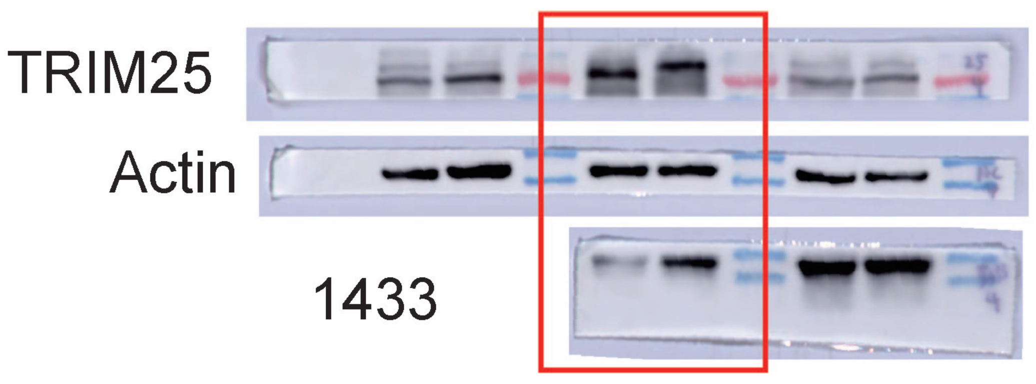

S3I

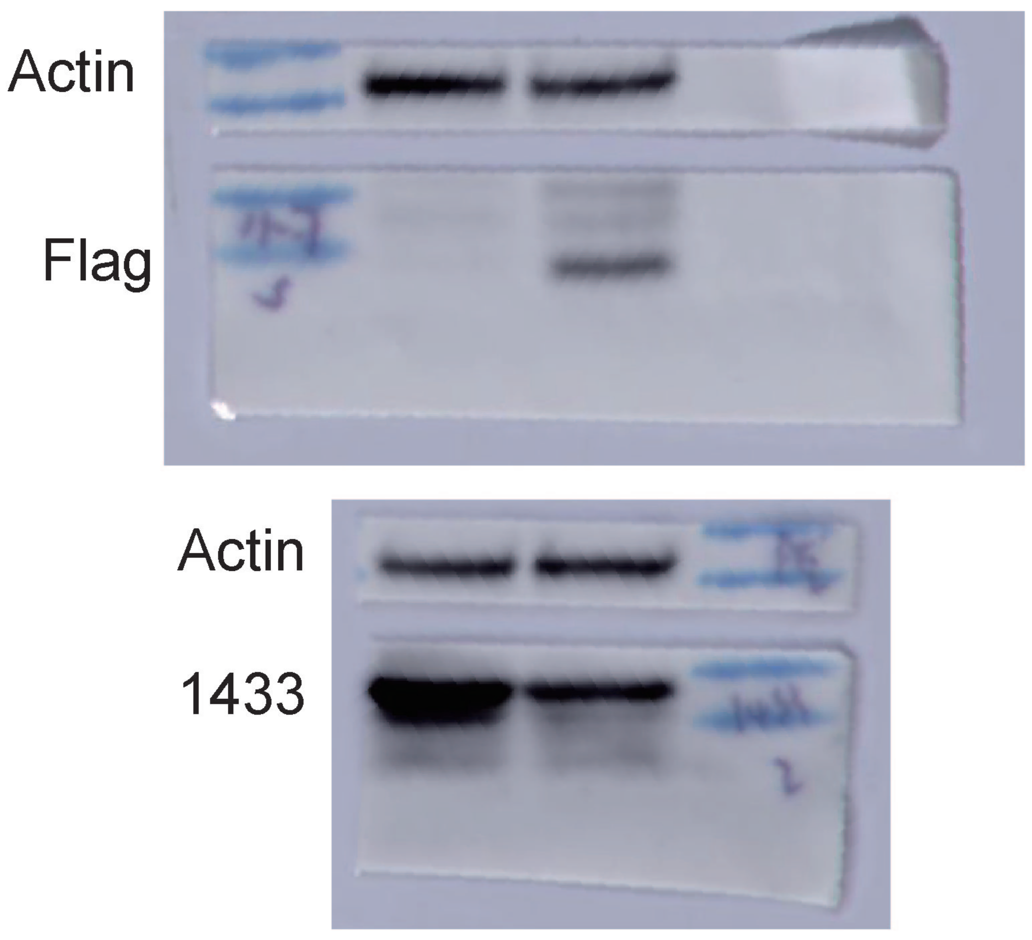

S3J

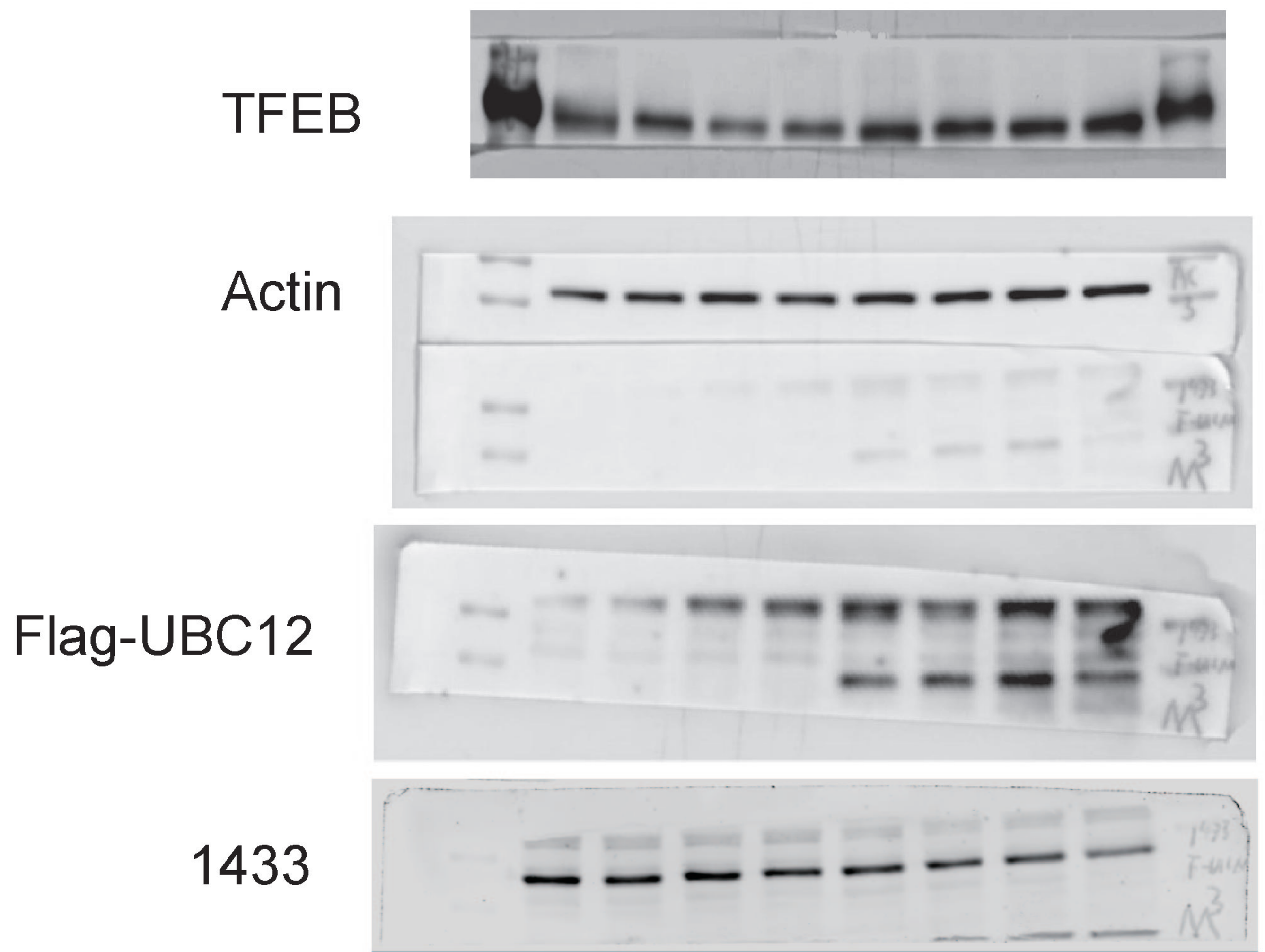

Supplement: Supplementary file 3 — Supplementary Material 3 [file 13046_2024_3085_MOESM3_ESM.pdf]
